# Controlling the thermoelectric properties of organo-metallic coordination polymers through backbone geometry†

**DOI:** 10.1039/d3fd00139c

**Published:** 2023-09-07

**Authors:** Zilu Liu, Md Azimul Haque, Chris N. Savory, Tianjun Liu, Satoru Matsuishi, Oliver Fenwick, David O. Scanlon, Martijn A. Zwijnenburg, Derya Baran, Bob C. Schroeder

**Affiliations:** a Department of Chemistry, University College London London WC1H 0AJ UK b.c.schroeder@ucl.ac.uk; b King Abdullah University of Science and Technology (KAUST), Physical Sciences and Engineering Division (PSE), KAUST Solar Center (KSC) 23955 Thuwal Saudi Arabia derya.baran@kaust.edu.sa; c Thomas Young Centre, University College London London WC1E 6BT UK; d School of Engineering and Materials Science, Queen Mary University of London London E1 4NS UK; e International Center for Materials Nanoarchitectonics (WPI-MANA), National Institute for Materials Science (NIMS) 1-1 Namiki, Tsukuba Ibaraki 305-0044 Japan; f Materials Research Center for Element Strategy, Tokyo Institute of Technology 4259 Nagatsuta-cho, Midori-ku Yokohama 226-8503 Japan; g Diamond Light Source Ltd., Diamond House, Harwell Science and Innovation Campus Didcot Oxfordshire OX11 0DE UK

## Abstract

Poly(nickel-benzene-1,2,4,5-tetrakis(thiolate)) (Ni-btt), an organometallic coordination polymer (OMCP) characterized by the coordination between benzene-1,2,4,5-tetrakis(thiolate) (btt) and Ni^2+^ ions, has been recognized as a promising p-type thermoelectric material. In this study, we employed a constitutional isomer based on benzene-1,2,3,4-tetrakis(thiolate) (ibtt) to generate the corresponding isomeric polymer, poly(nickel-benzene-1,2,3,4-tetrakis(thiolate)) (Ni-ibtt). Comparative analysis of Ni-ibtt and Ni-btt reveals several common infrared (IR) and Raman features attributed to their similar square-planar nickel–sulfur (Ni–S) coordination. Nevertheless, these two polymer isomers exhibit substantially different backbone geometries. Ni-btt possesses a linear backbone, whereas Ni-ibtt exhibits a more undulating, zig-zag-like structure. Consequently, Ni-ibtt demonstrates slightly higher solubility and an increased bandgap in comparison to Ni-btt. The most noteworthy dissimilarity, however, manifests in their thermoelectric properties. While Ni-btt exhibits p-type behavior, Ni-ibtt demonstrates n-type carrier characteristics. This intriguing divergence prompted further investigation into the influence of OMCP backbone geometry on the electronic structure and, particularly, the thermoelectric properties of these materials.

## Abstract 2

The thermoelectric properties of organometallic coordination polymers (OMCPs) are influenced by the geometry of their backbone, thus underscoring the intricate nature of the structural elements to consider when designing novel thermoelectric OMCPs.

## Graphical abstract

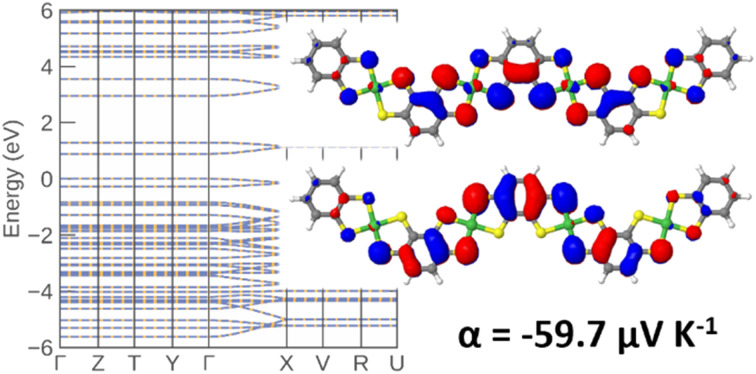

## Introduction

Organo-metallic coordination polymers (OMCPs) represent a fascinating class of hybrid materials that have attracted significant attention in recent years due to their unique structural and electronic properties.^1,2^ These materials consist of metal cations coordinated to organic ligands, thus forming extended networks. The combination of metal ions and organic ligands allows for the design and synthesis of materials with tailored properties. Metal-tetrathiolate (MS_4_) coordination systems, in particular, have garnered significant attention in recent years due to their self-assembly, high electrical conductance, metallic behaviour, porosity, and electrocatalytic activity.^3–8^ In addition, the ease of synthesis facilitated by employing polyfunctionalised aromatic ligands, such as 1,4-benzenedithiol, benzenehexathiol, triphenylenehexathiol or perthiolated coronene, has led to the production of a wide range of OMCPs with intriguing thermoelectric properties.^9–12^ Poly(nickel-benzene-1,2,4,5-tetrakis(thiolate)) (Ni-btt) stands out compared to other one-dimensional ladder-type polymers, *e.g.* poly(nickel-ethylenetetrathiolate) (Ni-ett), as Ni-btt displays p-type character, contrary to the commonly encountered n-type thermoelectric characteristics in Ni-based OMCPs.^13^

To optimise the thermoelectric properties of OMCPs further, significant efforts have been dedicated to material synthesis, batch-to-batch reproducibility, morphology control, and processability.^13–17^ The primary focus was put on the synthetic conditions by exploring different transition-metal centres, adjusting the ligand-to-metal ratios, controlling oxidation levels, and utilising electrochemical synthesis. Additionally, different processing approaches, such as ball-milling, blending with polymer binders, and thermal annealing, have enabled the successful fabrication of OMCP films and their incorporation into thermoelectric generators.^17–19^

To gain further insights into the electronic structures and physical properties of the poly(M-ett) family and its derivatives, density functional theory (DFT) calculations have proven instrumental.^20–23^ DFT calculations have facilitated the understanding of structure–property relationships and provided a basis for initial molecular design considerations by exploring different combinations of metal centres and organic ligands. Despite these advancements, the number of structurally novel OMCPs remains limited, and significant synthetic efforts are necessary to experimentally determine their potential structure–property relationships. In a recent study, we reported the thermoelectric performance of two novel linear π–d conjugated organometallic coordination polymers: poly(nickel-[2,2′-bi(1,3-dithiolylidene)]4,4′,5,5′-tetrakis(thiolate)) (Ni-diett) and poly(nickel-benzene-1,2,4,5-tetrakis(thiolate)) (Ni-btt), along with Ni-ett.^13^

Efforts to enhance the processability of organometallic coordination polymers (OMCPs) have become a significant focus of interest. One potential strategy to achieve this goal involves reducing molecular order through ligand design, which can render the polymer more soluble. In this study, we address this objective by synthesising a structural isomer of the btt ligand, namely benzene-1,2,3,4-tetrakis(thiolate) (ibtt). The ibtt ligand differs from the previously studied *D*_2h_-symmetric btt ligand in its *C*_2v_ symmetry and as such should reduce molecular order and thereby enhance solubility. Our study encompasses the preparation, structural characterization, and thermoelectric measurements of the OMCPs incorporating these two isomeric ligands, focusing on MS_4_ complexes with high structural integrity and a square-planar coordination.

In contrast to the previously reported p-type behaviour of Ni-btt, our investigations reveal that Ni-ibtt exhibits n-type characteristics. It displayed a moderate electrical conductivity (*σ*) on the order of 10^−2^ S cm^−1^ and an encouraging Seebeck coefficient (−60 μV K^−1^). Spectroscopic characterization techniques, including UV-visible, FTIR, and Raman spectroscopy, were employed to analyse Ni-ibtt and Ni-btt, as well as a series of intermediate compositions, Ni(ibtt)*_x_*(btt)_1−_*_x_*, to gain a deeper understanding of the underlying structure–property relationships.

Our work unveils the profound impact that modest structural differences in the organic ligands can have on the overall thermoelectric properties of the resulting OMCPs. Through a combination of experimental characterization and theoretical simulations, we provide valuable insights into the tuning of thermoelectric properties by altering the ratio of ligand isomers. Additionally, this work reports the synthesis of a metal-ibtt coordination polymer and its corresponding thermoelectric performance. Overall, our findings contribute to a deeper understanding of the relationship between organic ligand structure and thermoelectric properties in OMCPs.

## Results and discussion

### Synthesis

**Fig. 1:**
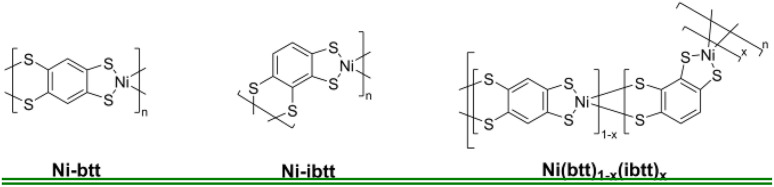
Chemical structures of the OMCPs incorporating different ligand isomers.

The synthesis of 1,2,4,5-tetrakis(isopropylthio)benzene (TIB) as a precursor for the preparation of Ni-btt [btt = benzene-1,2,4,5-tetrakis(thiolate)] followed the same method as described in our previous report.^13^ The synthesis of Ni-ibtt and Ni(ibtt)*_x_*(btt)_1−_*_x_* was conducted using a slightly adapted procedure, employing 1,2,3,4-tetrakis(isopropylthio)benzene (iTIB) as the OMCP precursor ligand. The detailed synthetic procedures are provided in the ESI† and the chemical structures of the various OMCPs are depicted in Fig. 1. The different samples were purified *via* Soxhlet extraction, washing the crude OMCP with deionized water and methanol for 24 hours each.

### Compositional and structural characterisation

**Fig. 2:**
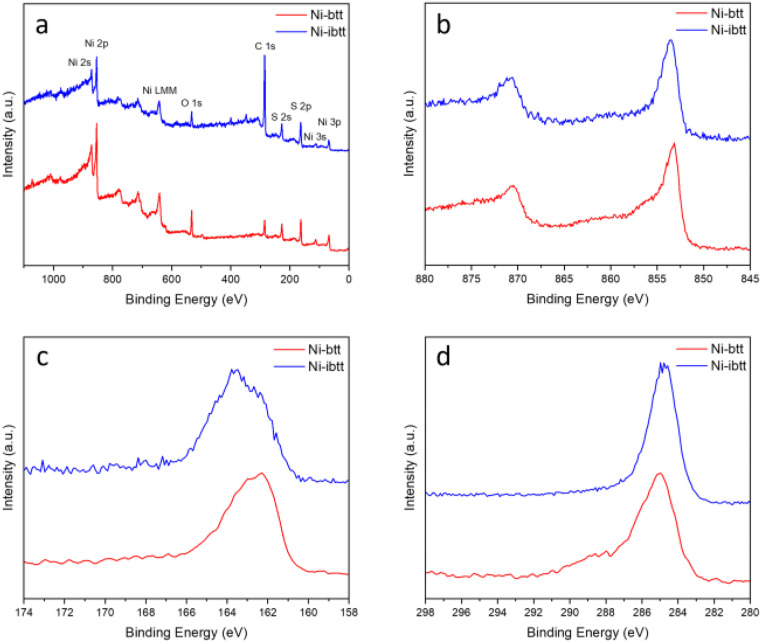
XPS spectra of Ni-btt and Ni-ibtt: (a) survey scan, (b) Ni 2p, (c) S 2p and (d) C 1s.

To analyse the chemical composition of the new Ni-ibtt, X-ray photoelectron spectroscopy (XPS) was conducted (Fig. 2 and S5†) and the data compared to the previously characterised Ni-btt. The survey spectra confirmed the presence of Ni, S, and C as the primary constituent elements (other than hydrogen, which our XPS system cannot detect). The Ni 2p photoemission spectrum of Ni-ibtt exhibited two peaks at binding energies of 853 and 873 eV, corresponding to the Ni 2p_3/2_ and Ni 2p_1/2_ spin–orbit splitting, respectively. These observations indicate the presence of a single type of Ni^2+^ species as the central metal. The S 2p XPS narrow-range scan with fitting reveals peaks at binding energies of 161.8 and 163 eV, which were attributed to Ni-thiolate complexation. In line with previous observations, neither the XPS energy survey spectrum, nor the Na 1s core level XPS spectrum (Fig. S6†) provided any evidence for the presence of residual Na from the synthesis, thus ruling out the possibility of excess Na acting as a counter anion in the Ni-ibtt OMCP.

To further assess the chemical composition, X-ray fluorescence (XRF) analysis was performed (Table S1†). The experimental S/Ni ratio determined by XRF for Ni-ibtt was 3.15, reasonably close to the theoretical S/Ni ratio of 4. We believe that the discrepancy between experimental and theoretical values can be explained by the poor solubility of Ni-ibtt and the challenging purification on the one hand, and by the likely presence of structural defects along the polymer backbones affecting the ratio on the other hand. Besides the S/Ni ratio, however, it is noteworthy that the XRF data confirmed the absence of any significant additional elements, Na or others, acting as potential counter cations in the Ni-ibtt sample, thus providing further evidence for the charge-neutral character of the polymer backbone. Prior studies on structurally similar MS_4_ and M(NH)_4_-based OMCPs described the presence of charge-neutral radicals and Raman spectroscopy experiments were performed to elucidate the distinct Raman signature of thiocarbonyl radicals (C=S˙).^24^

**Fig. 3:**
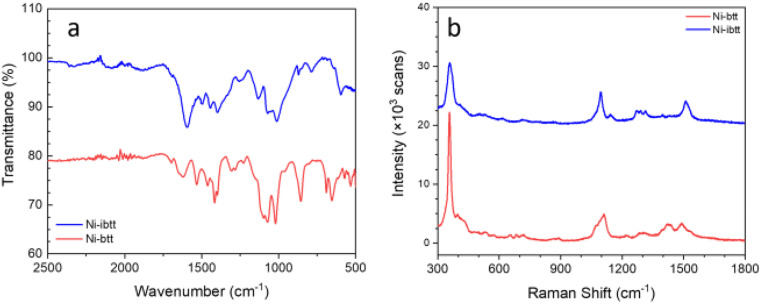
FTIR (a) and resonance Raman (728 nm excitation) (b) spectra of Ni-btt and Ni-ibtt after purification. The spectra have been stacked for clarity.

Attenuated total reflection Fourier transform infrared (ATR-FTIR) and resonance Raman spectroscopy were conducted on the organic ligands TIB and iTIB (Fig. S8†) and compared to the data collected for the corresponding OMCPs. In the FTIR spectrum of Ni-ibtt (Fig. 3a), the distinct C=S˙ stretching vibration modes were observed at 1133 cm^−1^, along with a split peak at 1071–1012 cm^−1^. The higher wavenumber range (1596–1398 cm^−1^) was attributed to the aromatic semicircular stretching and the ring quadrant stretching mode.

The Raman spectrum of Ni-ibtt exhibited several striking similarities to the Ni-btt spectrum, primarily due to the structural resemblance between the ibtt and btt ligands and the corresponding MS_4_ coordination bonds formed (Fig. 3b). The characteristic vibration mode at 357 cm^−1^ observed for both Ni-btt and Ni-ibtt is attributed to Ni–S coordination. Notably, Ni-ibtt displays a less intense peak from *ν*(Ni–S) compared to the series of aromatic peaks in the Raman spectrum, in contrast to Ni-btt. Considering the isomeric ligand, the presence of four adjacent C–S bonds in ibtt distorts the electric charge separation, resulting in a higher intrinsic dipole moment (*μ* = *q* × *d*, where *q* is the charge and *d* is the distance) with deviation from the centre of the benzene ring, in comparison to btt. Consequently, the coordination in Ni-ibtt leads to a considerable charge separation, with the positive charge located on the Ni atom while the negative charge is delocalized over the organic ligand. Raman vibrational modes originating from highly polar moieties typically exhibit weaker intensities. In other words, the external electric field cannot induce a notable change in the dipole moment of Ni–S in Ni-ibtt compared to Ni-btt, hence the *ν*(Ni–S) peak is weaker in intensity.

The Raman spectra of both organic ligands, TIB and iTIB (Fig. S9†), differ slightly in the signals arising from their respective out-of-plane C–H bending vibrations (<1000 cm^−1^). These vibrations, however, were not observable in the Raman spectra of the two OMCPs and appeared in the FTIR spectra instead at 870 cm^−1^ for Ni-ibtt, with a stronger band at 857 cm^−1^ in the case of Ni-btt. The sharp peak at 1095 cm^−1^ observed in the Raman spectrum of Ni-ibtt is attributed to the *ν*(C=S˙) stretching vibration of the ligand, accompanied by a weaker, adjacent peak at 1141 cm^−1^.

### Polymer backbone geometry and solid-state packing

**Fig. 4:**
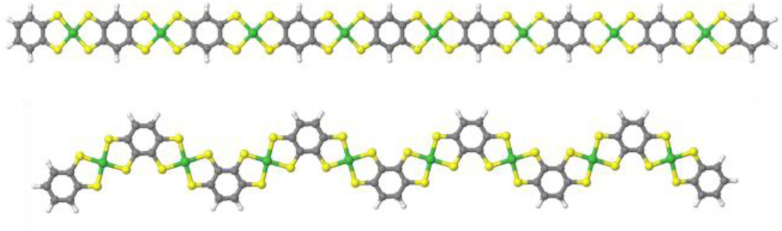
Minimum energy conformations obtained from DFT calculations for octamers of Ni-btt (top) and Ni-ibtt (bottom) with all metal–ligand complexes in *trans*-configuration.

After confirming the chemical composition of the novel Ni-ibtt, our focus shifted towards differences in the ‘shape’ of the polymers and their solid-state packing. Based on previous observations in the field of organic semiconducting polymers,^25,26^ the assumption was that the ibtt ligand with its *C*_2v_ symmetry would disrupt the polymer packing in the solid state and hence increase the solubility and processability of the resulting Ni-ibtt compared to Ni-btt. To investigate the effect of symmetry on OMCP backbone geometry, density functional theory (DFT) calculations were conducted on oligomeric models of Ni-btt and Ni-ibtt, discussed in more detail below, and the optimised molecular geometries are shown in Fig. 4.

Like Ni-btt, the Ni atoms in Ni-ibtt participate in the formation of square-planar complexes with the iTIB ligand. Due to the higher symmetry of the TIB ligand, the resulting Ni-btt OMCP has a linear polymer backbone. In Ni-ibtt, however, the lower *C*_2v_ symmetry of the organic ligand allows for the formation of either *cis*- or *trans*-configurations along the polymer backbone, leading to a wavy or zig-zagged backbone (Fig. 4). To facilitate the DFT calculations, we assumed a 100% *trans*-arrangement in Ni-ibtt. During the synthesis, however, no efforts were made to control the orientation of the organic ligands relative to each other, and it should be assumed that their arrangement is random in the synthesised Ni-ibtt. Despite this increased disorder along the polymer backbone, the planar geometry of the NiS_4_ complex ensures that the overall planarity of the OMCP is not affected. In the unlikely event that all metal–ligand complexes would arrange in a *cis*-configuration in the Ni-ibtt, it would be possible for the chain ends to link up and form a cyclic oligomer (Fig. S13†). While the formation of cyclic oligomers comprising [Ni_6_L_6_] is conceptually possible, no experimental evidence confirming their existence was found, and we will therefore disregard this molecular arrangement from the discussion.

To assess the crystallinity of the OMCP based on the isomeric ligands, powder X-ray diffraction (XRD) spectra were recorded for both materials. In comparison to the linear Ni-btt, Ni-ibtt exhibited two broad peaks at 5.9° and 12.1° that were similar in position to the peaks in the former. However, an additional peak at 3.8° was observed in Ni-ibtt, indicating a wider *d*-spacing of 10.7 Å, resulting from its wavy and less ordered backbone (Fig. S14†).

### Thermoelectric characterization

Before studying the thermoelectric properties of Ni-ibtt, it was important to evaluate its thermal stability through thermogravimetric analysis (TGA) (Fig. S15†). In comparison to Ni-btt, Ni-ibtt exhibited a lower thermal stability and gradually started degrading from 150 °C onwards. Moreover, the degradation of Ni-ibtt was slower compared to Ni-btt, which displayed a sharp weight loss at approximately 300 °C. Despite the decomposition temperature of Ni-ibtt being relatively elevated, thermal annealing of a Ni-ibtt pellet at 150 °C, under ambient conditions, led to partial surface oxidation as evidenced by XPS measurements (Fig. S7†). The S 2p peak at 168 eV is representative of sulfate contaminants in the sample. Notably, the oxidation was limited to the surface, and after sputtering 15 nm beneath the surface, the XPS spectrum revealed again a pristine S 2p peak without any discernible change indicative of oxidation.

**Fig. 5:**
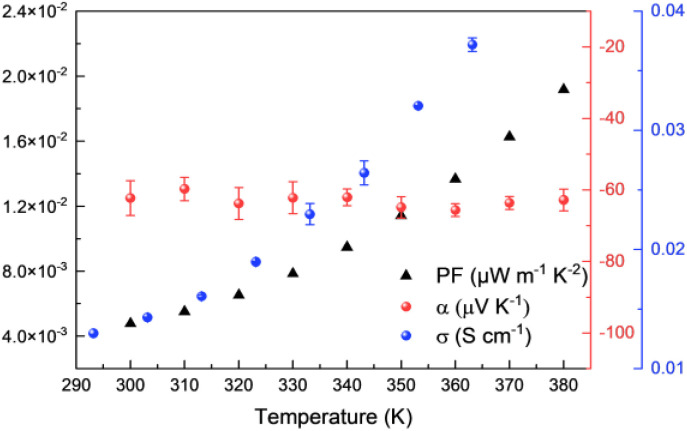
Thermoelectric properties of Ni-ibtt: electrical conductivities (blue dots), Seebeck coefficients (red dots) and power factors (black triangles).

Intrinsically air-stable n-type organic thermoelectric materials are scarce, and while many OMCPs previously demonstrated n-type behaviour, Ni-btt was one of the first materials to show p-type behaviour instead, as a consequence of the chemical structure of the ligand. Considering that Ni-ibtt is a constitutional isomer of Ni-btt, we were expecting the OMCP to display p-type thermoelectric characteristics. The thermoelectric properties of Ni-ibtt are depicted in Fig. 5, demonstrating, contrary to our hypothesis, that Ni-ibtt is another air-stable n-type OMCP with a negative Seebeck coefficient, similar to the widely reported Ni-ett.

Ni-ibtt exhibited a relatively stable Seebeck coefficient of around −60 μV K^−1^ in the studied temperature range. The profound effect of organic ligand isomerism on the nature of transported charges was unexpected. To date only one report explored Ni-ibtt in an all-trans conformation for its thermoelectric potential, relying on DFT calculations.^23^ The thermoelectric properties were computed both for p- and n-type performance, yet no conclusion with regards to the Seebeck was drawn. However, it was stated that the orbital conjugation between the sulfur atoms in the para-positions and the bridging C–C bonds would be the only hole-conducting channel and that therefore Ni-ibtt would be “detrimental for hole transport”,^23^ a finding that is consistent with our observation of n-type behaviour in Ni-ibtt and which could explain why Ni-btt and Ni-ibtt display opposing behaviour.

To gain a deeper understanding of the electronic structure of the isomeric OMCPs, DFT calculations were performed on both finite cluster (oligomers) and periodic (infinitely repeating chains) models of isolated Ni-btt and Ni-ibtt polymers. The cluster DFT calculations, using the hybrid B3LYP functional,^27–30^ on the tetrameric oligomeric models of Ni-btt and Ni-ibtt (see Fig. S19†) predict that while the electronic ground state of Ni-btt is a spin-polarised open-shell singlet, the electronic ground state of Ni-ibtt is a closed-shell singlet. Similarly, in the periodic DFT calculations, which use the hybrid HSE06 functional,^31,32^ the ground-state configuration of Ni-btt was found to be an open-shell singlet and that of Ni-ibtt a closed-shell singlet. The predicted closed-shell character of Ni-ibtt was experimentally confirmed by electron paramagnetic resonance (EPR) and superconducting quantum interference device (SQUID) measurements (Fig. S16 to S18†). In the EPR spectra, two main types of signals were observed: a broad low-field signal and a narrow central-field signal. The former can be identified as a high-spin (triplet) state of Ni^2+^, whereas the latter originates from a low spin Ni^3+^. However, the concentration of unpaired electrons is very low, indicating that Ni-ibtt is intrinsically nonmagnetic with a low spin state of Ni^2+^ (*S* = 0).

When visualising the highest occupied and lowest unoccupied molecular orbitals (HOMO and LUMO) of the Ni-ibtt tetramer (see Fig. S20),† there is evidence of “cross-conjugation”. The contribution of the atomic orbitals of the two sulfur atoms along one diagonal of the NiS_4_ coordination environments is larger than that of the sulfur atoms along the other diagonal. In contrast, the contribution of the sulfur atoms around the nickel cations in Ni-btt to the highest singly occupied and lowest singly unoccupied orbitals (SHOMO/SLUMO) is much more symmetric. The difference is that in btt each sulfur atom of the ligand coordinating a nickel cation on one side of the ligand is always para with respect to a sulfur atom of the same ligand coordinating another nickel cation, while in ibtt they are a combination of *ortho*, *meta* and *para*. The calculations on the oligomers, finally, also suggest that Ni-btt has a deeper lowest unoccupied orbital/more positive electron affinity than Ni-ibtt, and hence would be easier to reduce and n-dope than Ni-ibtt, while both materials are predicted to have similar HOMO/SHOMO and ionisation potential values, and hence should be comparably easy to p-dope.

**Fig. 6:**
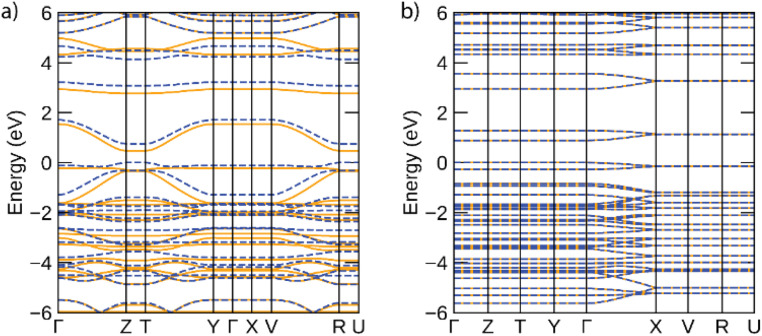
Electronic band structures of (a) Ni-btt and (b) 100%-*trans*Ni-ibtt, calculated using the HSE06 functional. Spin-up bands are depicted by solid orange lines, spin-down by dashed blue lines, and the valence band maximum is set to 0 eV. This figure was plotted using the sumo package.^33^

The band structures predicted for infinite Ni-btt and Ni-ibtt polymers in the periodic calculations are plotted in Fig. 6. The electronic structures of both coordination polymers showed them to be narrow-gap semiconductors, with gaps of 0.47 eV for Ni-btt and 0.88 eV for Ni-ibtt; the wider gap of Ni-ibtt likely results from the narrower bandwidths of both conduction and valence bands. In both electronic structures, in accordance with the modelled cells preventing inter-chain interactions, band dispersion is only seen along reciprocal lines corresponding to the polymer chain direction (*e.g. Γ* to *Z* for Ni-btt and *Γ* to *X* for Ni-ibtt). In Ni-btt, the conduction band is highly disperse in both spin channels – using a parabolic fit to the band edge gives an effective mass of 0.11 *m*_0_ (units of electron rest mass). The valence band shows significant dependence on the spin channel, with a heavy hole state in the spin up, but high local curvature at the valence band maximum in the spin down – the hole effective mass at the band edge is 0.16 *m*_0_, comparable with the conduction band, and consistent with the high p-type conductivities observed. By comparison, both conduction and valence bands of Ni-ibtt show weaker dispersion than those of Ni-btt, in line with the cross conjugation observed in the oligomeric calculations, and higher effective masses of 0.42 *m*_0_ and 0.70 *m*_0_, respectively. In all, the partial cross-conjugation in the all-*trans*Ni-ibtt (as seen in the partial charge density in ESI Fig. S21,† and as discussed also observed in the oligomeric calculations) impacts band dispersion in comparison with the wholly linear Ni-btt, and appears to lead to weaker conductivities, but higher Seebeck coefficient in that system.

As discussed above, the results of the calculations suggest, in contrast to experiment, that Ni-btt should behave as n-type and Ni-ibtt as p-type, other than perhaps the prediction of the significantly lower effective mass of electrons in the conduction band than holes in the valence band for Ni-ibtt, which should make the former considerably more mobile than the latter, if present. It is important, however, to remember that these calculations are for the intrinsic materials and do not consider the effect of defects in the materials and how these might result in (self-)doping. The defect chemistry of these materials, be it intrinsic or extrinsic, as the result of ppm/ppb-level impurities present from synthesis, is poorly understood and likely the p-type character of Ni-btt and the n-type character of Ni-ibtt find their origin in differences in their defect chemistry.

Further differences between the two OMCPs were observed in their respective electric conductivities. Similar to Ni-btt, Ni-ibtt displayed thermally activated transport, gradually increasing from 1.4 × 10^−2^ S cm^−1^ at 303 K to 3.7 × 10^−2^ S cm^−1^ at 363 K. It is noteworthy, however, that the measured electrical conductivities are two orders of magnitude lower in Ni-ibtt compared to Ni-btt. The electrical conductivity of a coordination polymer depends on numerous factors, such as the extent of conjugation length, intra- and intermolecular orbital overlap, and overall crystallinity or molecular order of the OMCP. The most striking difference between the two materials is their molecular structure and how the Ni-ibtt polymer was designed with the aim to disrupt molecular packing. Whilst the XRD provided some evidence for increased *d*-spacing and molecular disruption, the lower electrical conductivities are most likely a direct consequence of poorer molecular order and reduced interchain π-stacking in the zig-zagged Ni-ibtt, compared to the linear Ni-btt. Another contributor to the lower observed mobility could be the presence of structural defects in Ni-ibtt, which has been demonstrated in the past to be detrimental to electrical properties.^34^ As outlined earlier, partial oxidation of the sulfur in the OMCP backbone cannot be excluded and could introduce additional chemical defect sites, impeding efficient charge transport.

To investigate the effect of the isomeric ligands on the thermoelectric properties further, we synthesised two intermediate OMCP compositions, Ni(ibtt)_0.5_(btt)_0.5_ and Ni(ibtt)_0.2_(btt)_0.8_. The detailed synthetic procedures and full material characterisation are provided in the ESI.† The thermoelectric properties of all four OMCP materials are summarised in Table 1. Of all four materials, Ni(ibtt)_0.5_(btt)_0.5_ should experience the largest interchain disorder as the introduction of the more linear btt ligand will prevent any longer-range order in the polymer, due to the geometry mismatch between the two isomeric ligands. This assumption is supported by the much lower electrical conductivity recorded for Ni(ibtt)_0.5_(btt)_0.5_. As the interchain order is increased by introducing more btt, the electrical conductivity increases by two orders of magnitude for Ni(ibtt)_0.2_(btt)_0.8_, approaching the conductivity of Ni-btt. The most striking impact of ligand mixing in the OMCP however was seen on the Seebeck coefficient. Neither of the two mixed-ligand OMCPs displayed n-type characteristics, which was reserved to Ni-ibtt. For both Ni(ibtt)_0.5_(btt)_0.5_ and Ni(ibtt)_0.2_(btt)_0.8_, the measured Seebeck coefficients were positive and of a similar magnitude as for Ni-btt, highlighting the importance of carefully considering the ligand composition in these OMCPs. As outlined previously, Ni-ibtt is a poor hole-transporting material and therefore the introduction of a good hole-transporting material, like the linear btt ligand, was expected to have a tremendous impact on the mixed OMCP transport properties.

**Table 1:** Thermoelectric properties of Ni-btt, Ni-ibtt and Ni(ibtt)*_x_*(btt)_1−_*_x_* at 310 K

Sample	*σ* (S cm^−1^)	*α* (μV K^−1^)	PF (μW m^−1^ K^−2^)
Ni-ibtt	1.61 × 10^−2^	−59.7	5.11 × 10^−3^
Ni(ibtt)0.5(btt)0.5	2.54 × 10^−3^	12.8	4.24 × 10^−5^
Ni(ibtt)0.2(btt)0.8	1.21 × 10^−1^	17.2	3.52 × 10^−3^
Ni-btt	7.11	12.7	1.20 × 10^−1^

## Conclusions

In conclusion, we have synthesized a series of one-dimensional OMCPs with varying degrees of backbone linearity by introducing different constitutional isomers into the polymer backbone. The difference in bridging ligands gave rise to Ni-ibtt, with a more disordered zig-zagged backbone, and Ni-btt with a rigid and linear geometry. Through careful characterisation we were able to determine the chemical composition of both isomeric organometallic coordination polymers and confirm the charge-neutral character of the polymer backbone. When assessing the thermoelectric properties of the novel OMCPs, we found that the wavy, less ordered backbone of Ni-ibtt yielded a much lower electrical conductivity (1.61 × 10^−2^), while at the same time displaying a negative Seebeck coefficient (−59.7 μV K^−1^). By moderating the ratio of btt/ibtt ligands in the OMCPs and subsequently the backbone geometry and molecular order in the materials, we were able to gain control over the thermoelectric properties and moderate both the charge transport and the thermovoltage. To the best of our knowledge, this is the first experimental demonstration of the profound impact of OMCP backbone geometry on thermoelectric properties, thus highlighting the complexity of various structural factors to consider when designing and optimizing new thermoelectric OMCPs.

## Author contributions

ZL and BCS contributed to conception, design, data acquisition (synthesis, FTIR, Raman, UV-vis, TE characterisation). MAH, TL, OF and DB recorded the thermoelectric properties. CNS, DOS and MAZ performed the DFT calculations. SM conducted the SQUID and EPR measurements. All authors contributed to data analysis, drafted, and critically revised the article. All authors gave their final approval and agree to be accountable for all aspects of the work.

## Conflicts of interest

There are no conflicts to declare.

## Supplementary Material

FD-250-D3FD00139C-s001

FD-250-D3FD00139C-s002
