# Supplementary material for: Controlling the thermoelectric properties of organo-metallic coordination polymers through backbone geometry
Source: Faraday Discuss. 2023 Sep 7;250:377–89. doi: 10.1039/d3fd00139c (PMC10926974; doi:10.1039/d3fd00139c)
Supplement: FD-250-D3FD00139C-s002 [file FD-250-D3FD00139C-s002.pdf]

Electronic Supplementary Material (ESI) for Faraday Discussions.
This journal is © The Royal Society of Chemistry 2023

Supplementary Information

Controlling the thermoelectric properties of organo-metallic coordination polymers through backbone geometry

*Zilu Liu, Md Azimul Haque, Chris N. Savory, Tianjun Liu, Satoru Matsuishi, Oliver Fenwick, David O. Scanlon, Martijn A. Zwijnenburg, Derya Baran * and Bob C. Schroeder **

Email: derya.baran@kaust.edu.sa ; b.c.schroeder@ucl.ac.uk

Table of Contents

General Experimental 2
Synthetic procedures 3
1,2,3,4-Tetrakis(isopropylthio)benzene (iTIB) 3
Poly[nickel-benzene-1,2,3,4-tetrakis(thiolate)] (Ni-ibtt) 3
1,2,3,4-tetrakis(methylthio)benzene (1) 3
Intermediate compositions $Ni(ibtt)_x(btt)_{1-x}$ 4
X-ray photoelectron spectroscopy 7
X-ray fluorescence analysis 9
Fourier transform infrared and Raman spectroscopy 10
Powder X-ray diffraction 13
Thermogravimetric analysis 13
Electron paramagnetic resonance 14
Density functional theory 16
Thermoelectric characterisation 19
References 20

General Experimental

All reagents and solvents were obtained from commercial suppliers and used without further purification. Reactions were carried out under nitrogen atmosphere using standard Schlenck techniques and reported yields refer to purified and spectroscopically pure compounds, unless otherwise stated. Concentration under reduced pressure was performed by rotary evaporation (25 - 40 °C) at an appropriate pressure. Thin layer chromatography (TLC) was performed using Merck Si60 F254 pre-coated TLC aluminium plates and flash column chromatography was performed using Merck Geduran Si 60 silica gel (40–63 µm particle size). ^{1}H and ^{13}C nuclear magnetic resonance (NMR) spectra were recorded on a Bruker NMR spectrometer *Avance III 400* at 298 K. Chemical shifts (δ) are reported in parts per million (ppm), using the residual solvent peaks as internal standard. For ^{1}H-NMR (δ): $CDCl_3$ 7.26. For ^{13}C-NMR (δ): $CDCl_3$ 77.16. Coupling constants (J) are given in Hertz (Hz) and spin multiplicities denoted as follows: s = singlet, d = doublet, hept = heptet. Mass spectrometry data was collected on a *Thermo Accela LC/LTQ* (EI) and *Waters LCT Premier QTOF* (ESI).

Bruker Platinum ATR with a single reflection diamond attachment was used to record Fourier-transform infrared spectra (FTIR). *Renishaw inVia* Raman spectrometer equipped with a *Leica DM LM* microscope was used to perform the Raman spectroscopy at room temperature under ambient conditions. A 785 nm diode laser was chosen as the excitation source for all the samples with a maximum power of 500 mW. Thermal stabilities were examined by a *TA instruments Q500 TGA* under nitrogen atmosphere over a range of 293 K to 1073 K at a constant heating rate of 20 K min^{-1}. Energy dispersive X-ray fluorescence (ED-XRF) data was collected by a *Malvern Panalytical Epsilon 4* spectrometer equipped with a silver anode X-ray tube. X-ray diffraction (XRD) was performed by a *STOE SEIFERT* diffractometer using Mo K_α (0.7093 Å) radiation with a detected angular range of $2° < 2\theta < 40°$ and a step size of 0.05° at 1 s per step. MDI Jada 6 software was used for the XRD data analysis. X-ray photoelectron spectroscopy (XPS) was performed by a *Thermo Scientific K-Alpha* spectrometer with a monochromated Aluminium (Al) K_α X-ray source (1486.7 eV). All XPS data was processed with the CasaXPS software and calibrated against the C 1s peak to 284.6 eV. EPR spectra were measured at 77 K using an EMX X-band (~10 GHz) spectrometer (Bruker, Germany) with a cylindrical microwave resonator and a liquid nitrogen Dewar. The concentration of unpaired electron was estimated by comparing the area of observed absorption peak with that of $CuSO_4 \times 5\ H_2O$ standard sample (Cu^{2+}, d9, S = 1/2). DC magnetization measurements were performed using a MPMS3 magnetometer (Quantum Design, USA) for a temperature of 2-300 K and magnetic field of 0 - 7 T. Electrical conductivity and Seebeck measurements were performed on OMCP pellets. The pellets were prepared by grounding the OMCP samples into fine powder with mortar and pestle before compressing the powder into round-shaped pellets (12.5 mm diameter; thickness between 0.8 – 1.2 mm) using KBr pellet dies applying a pressure of around 2.6 GPa. The thermoelectric characterisation was conducted on a *Netzsch SBA 548 Nemesis* thermoelectric set up under Helium environment using a four-probe technique and dual Peltier configuration, respectively.

Synthetic procedures

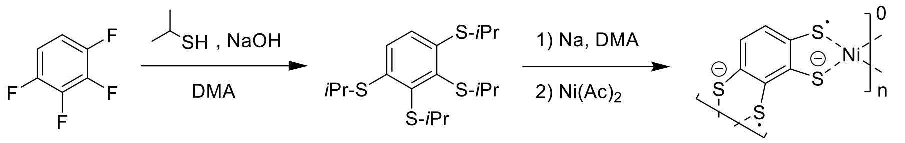

iTIB Ni-ibtt

1,2,3,4-Tetrakis(isopropylthio)benzene (iTIB)

1,2,3,4-tetrafluorobenzene (0.9 g, 6 mmol) and sodium hydroxide (1.83 g, 45.7 mmol) were dissolved in 20 mL of anhydrous *N,N*-dimethylacetamide (DMA) under an inert atmosphere (N_2), followed by the dropwise addition of 2-propanethiol (2.90 mL, 31.3 mmol) at room temperature. The solution was then refluxed at 100 °C for 2 h. Afterwards the solution was cooled to room temperature and 30 mL of brine was slowly added under stirring. The crude product was extracted with ethyl acetate (4 × 25 mL) and the combined organic layers filtered through a silica plug to remove residual DMA. After solvent evaporation, the product (iTIB) was recovered as a light-yellow oil (2.04 g, 91%). ^{1}H-NMR (400 Hz, $CDCl_3$, δ): 7.14 (s, 2H), 3.59 (hept, J=6.7 Hz, 2H), 3.42 (hept, J=6.7 Hz, 2H), 1.36 (d, J=6.7 Hz, 12H), 1.22 (d, J=6.7 Hz, 12H). ^{13}C-NMR (100 Hz, $CDCl_3$, δ): 141.7, 140.5, 127.2, 41.1, 36.6, 127.2, 23.2, 22.9. MS (EI): calcd. for $C_{18}H_{31}S_4$ $[M+H]^+$ 375.13, 376.13,377.13; obs. 375.13, 376.13, 377.13.

Poly[nickel-benzene-1,2,3,4-tetrakis(thiolate)] (Ni-ibtt)

iTIB (1.51 g, 4 mmol) was dissolved in anhydrous *N, N*-dimethylacetamide (75 mL) under inert atmosphere (N_2). Sodium pieces (5.2 g, 226 mmol) were then added to the solution and heated to 100 °C for 24 hours. Afterwards 15 mL of degassed and deionised water were added dropwise into the reaction slurry to yield an orange-brown solution. Nickel acetate tetrahydrate (1.02 g, 4 mmol) dissolved in 20 mL of degassed water was added dropwise over 15 minutes, during which the solution gradually turned deep black in colour. The mixture was further heated at 100 °C for another 24 hours. The crude black solid was collected by centrifugation and further purified by Soxhlet extraction with deionized water for 24 h, followed by methanol for 24 hours. The purified Ni-ibtt was dried for 24 hours in vacuum at 100 °C and recovered as a black powder (0.75g, 72%).

1,2,3,4-tetrakis(methylthio)benzene (1)

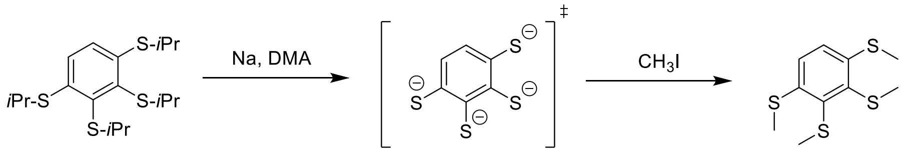

iTIB 1

To verify that the intermediate formed after sodium reduction was indeed 1,2,3,4-benzenetetrakis(thiolate), **iTIB** (110 mg, 0.294 mmol) was dissolved in 5 mL of anhydrous *N, N*-dimethylacetamide. Sodium pieces (380 mg, 16.5 mmol) were then added to the solution and

heated to 100 °C for 24 hours. Afterwards, the reaction was cooled to room temperature and quenched by the addition of methyl iodide (250 mg, 1.76 mmol). The crude product was extracted with ethyl acetate, washed with water, and dried over $MgSO_4$. After filtration, the organic solvent was removed, yielding 1,2,3,4-tetrakis(methyl)benzene **1** (63 mg, 0.24 mmol, 82%). ^{1}H-NMR (400 Hz, $CDCl_3$, δ): 7.06(s, 2H), 2.43(s, 6H), 2.41(s, 6H). ^{13}C-NMR (100 Hz, $CDCl_3$, δ):142.3, 139.3, 124.7, 20.0, 16.3. HRMS (ESI): calcd. for $C_{10}H_{15}S_4$ $[M+H]^+$ 263.0057; obs. 263.0058.

Intermediate compositions Ni(ibtt)$_x$(btt)$_{1-x}$

Ni(ibtt)$_{0.5}$(btt)$_{0.5}$ and Ni(ibtt)$_{0.2}$(btt)$_{0.8}$ were prepared following the same procedure as outlined above for Ni-ibtt. The molar ratios of the respective organic ligand precursors 1,2,4,5-tetrakis(isopropylthio)benzene (TIB) and 1,2,3,4-tetrakis(isopropylthio)benzene (iTIB) were adjusted accordingly.

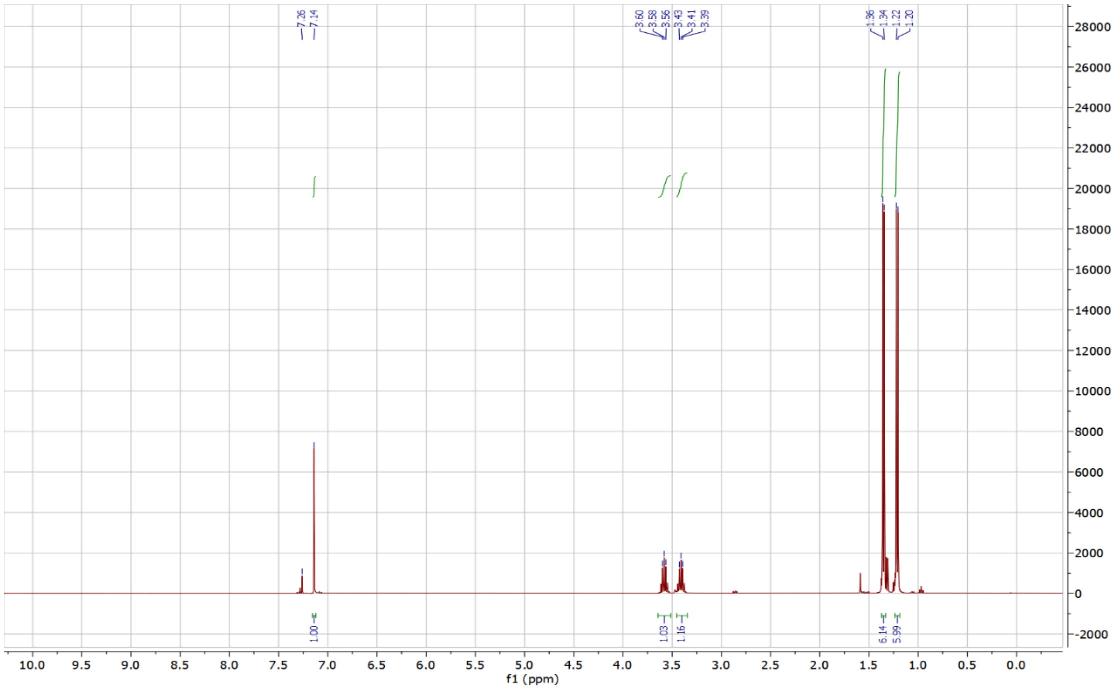

Figure S1. ^{1}H-NMR of **iTIB**.

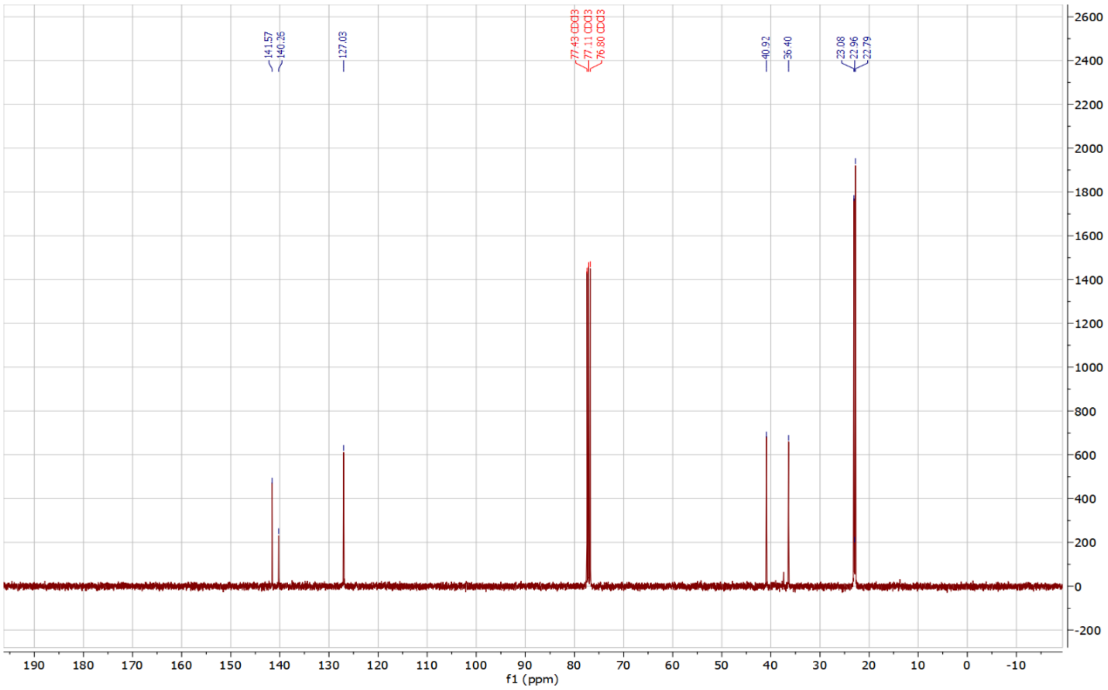

Figure S2. ^{13}C-NMR of **iTIB**.

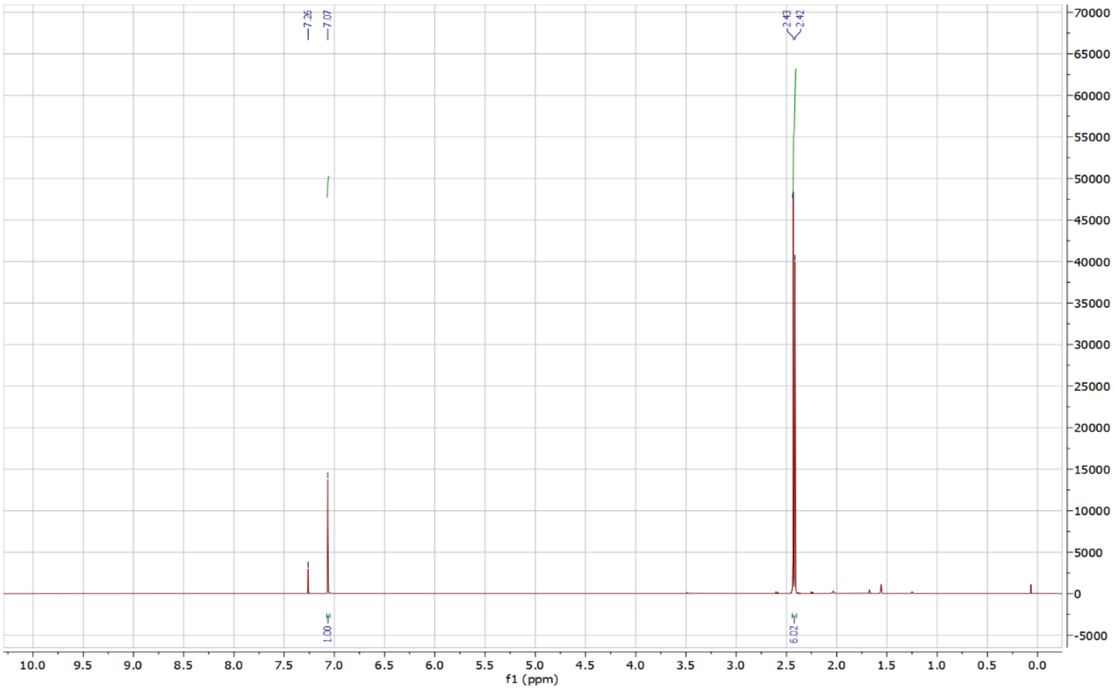

Figure S3. ^{1}H-NMR of compound **1**.

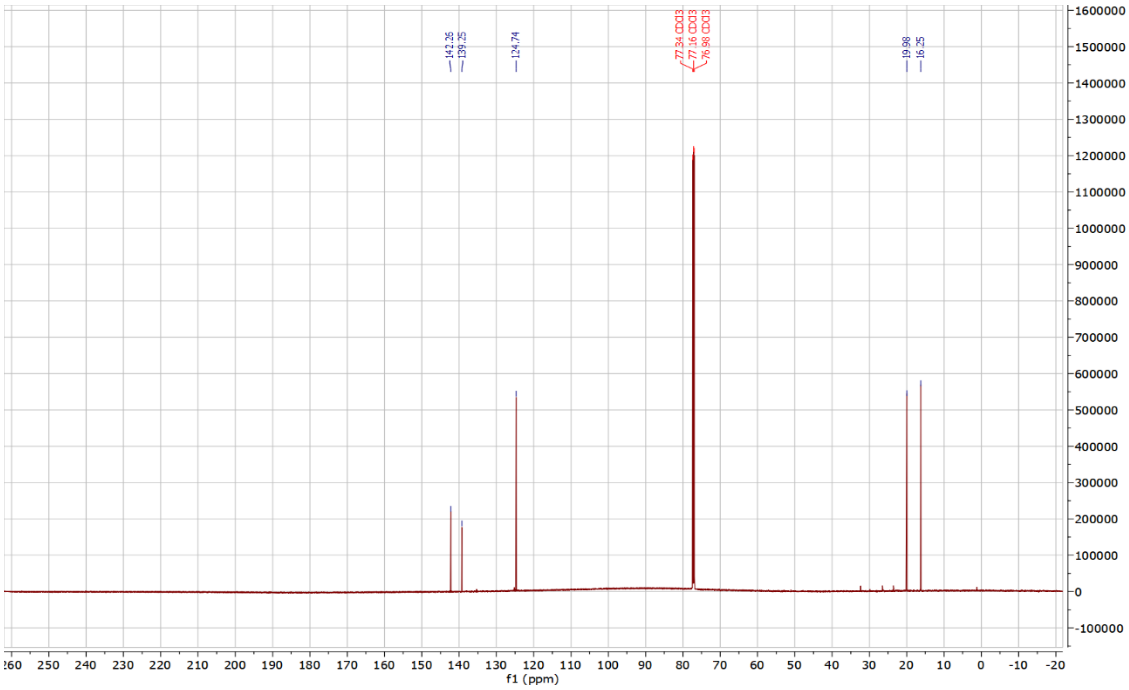

Figure S4. ^{13}C-NMR of compound **1**.

X-ray photoelectron spectroscopy

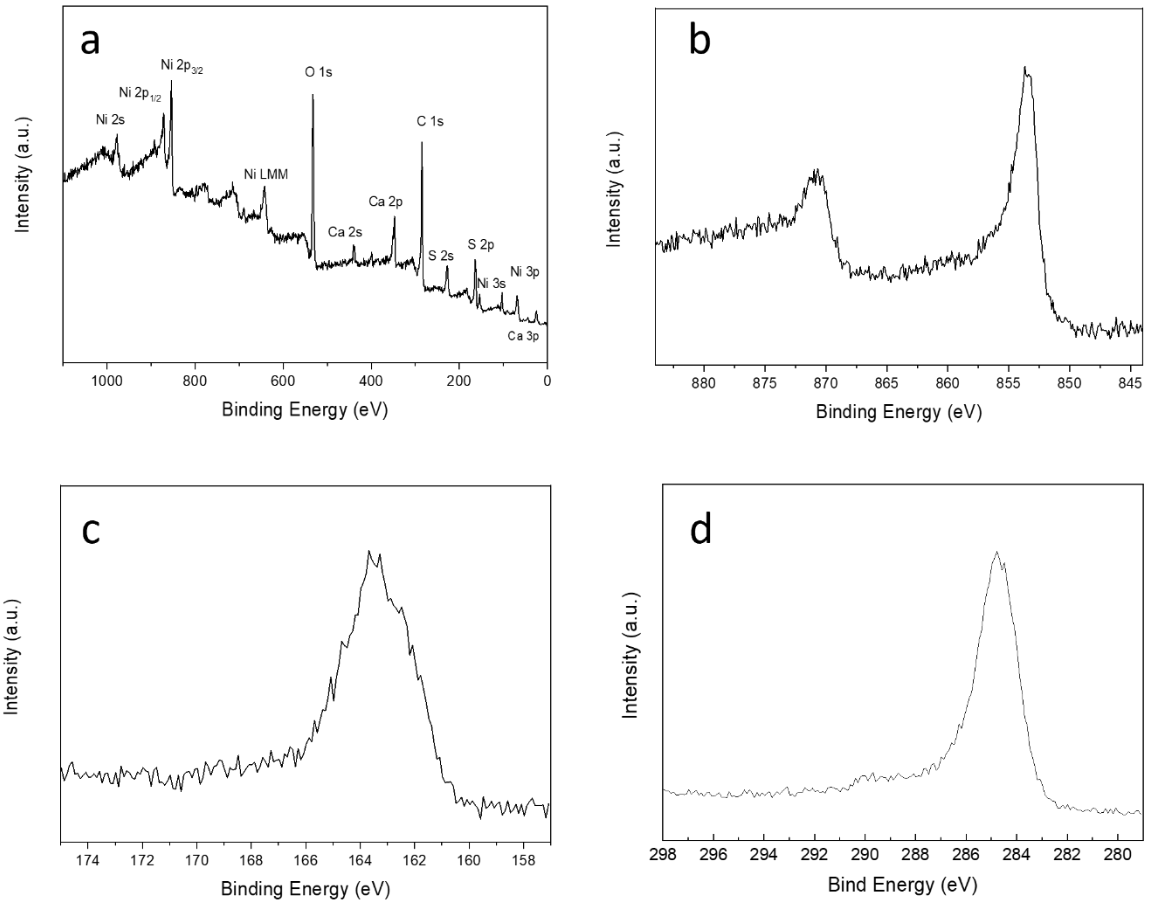

Figure S5. XPS spectra of Ni-(ibtt)$_{0.5}$(btt)$_{0.5}$, (a) survey scan, (b) Ni 2p, (c) S 2p and (d) C 1s.

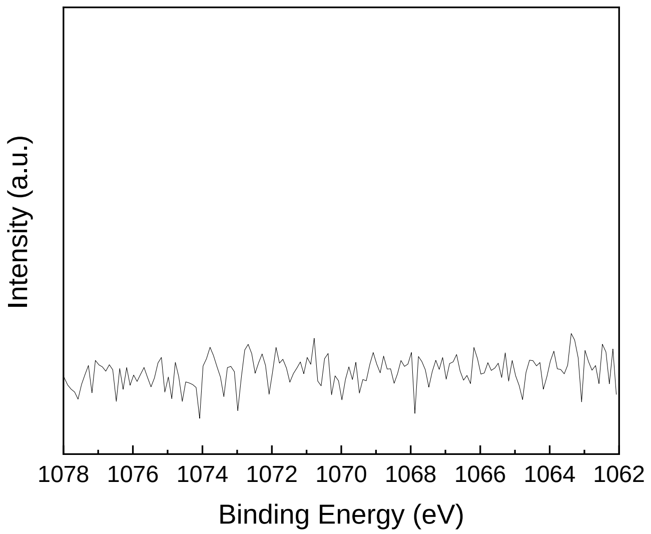

Figure S6. Na 1s core level XPS spectrum of Ni-(ibtt).

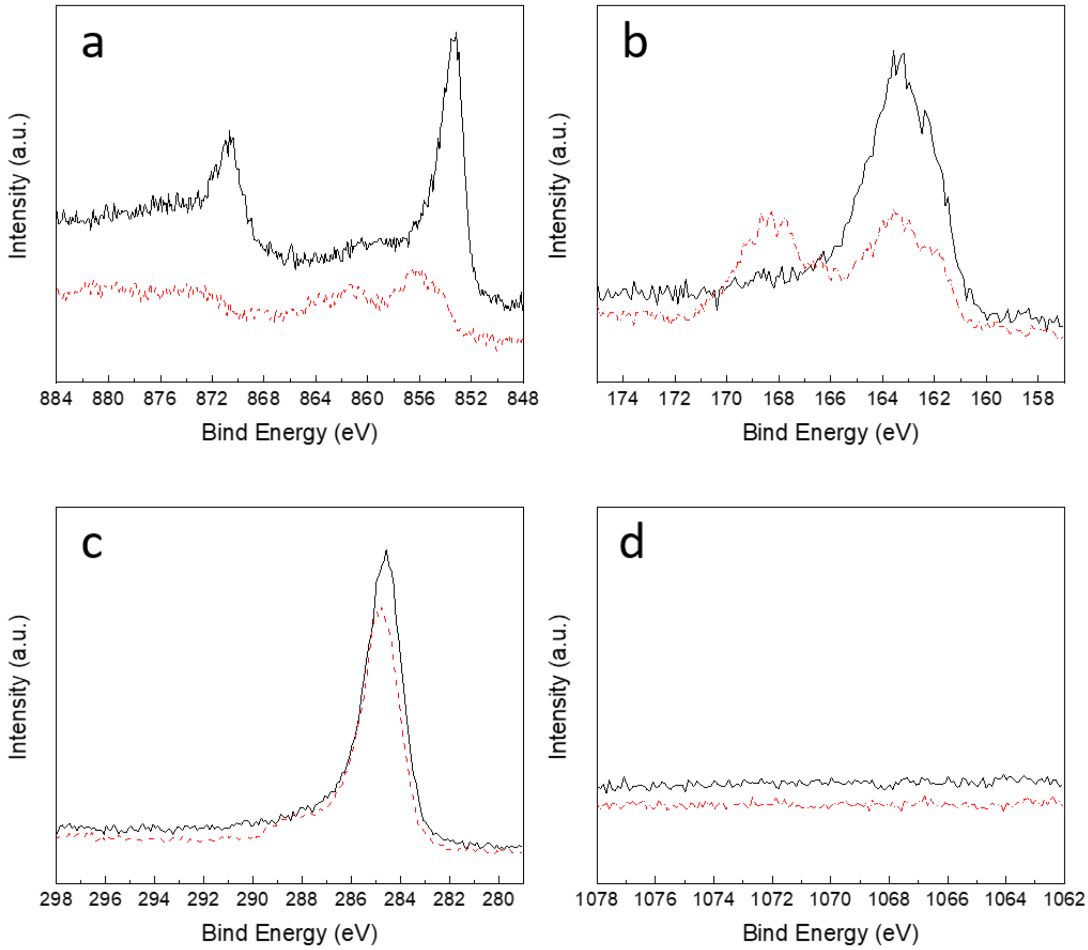

Figure S7. XPS spectra of the annealed pellet of Ni-ibtt on the surface (red dash) and 15 nm beneath (black solid): (a) Ni 2p, (b) S 2p, (c) C 1s and (d) Na 1s.

X-ray fluorescence analysis

Table S1: X-ray fluorescence analysis (XRF) of Ni-ibtt, Ni-btt, $Ni(ibtt)_{0.5}(btt)_{0.5}$ and $Ni_1(ibtt)_{0.2}(btt)_{0.8}$.

Ni-ibtt S/Ni = 3.15			Ni-btt S/Ni = 3.31			$Ni_1(ibtt)_{0.5}(btt)_{0.5}$ S/Ni = 3.61			$Ni_1(ibtt)_{0.2}(btt)_{0.8}$ S/Ni = 3.00		
Element	wt%	at%	Element	wt%	at%	Element	wt%	at%	Element	wt%	at%
**S**	**59.90%**	**74.80%**	**S**	**62.50%**	**74.30%**	**S**	**63.50%**	**77.50%**	**S**	**52. 8 %**	**68.50%**
**Ni**	**34.80%**	**23.70%**	**Ni**	**34.50%**	**22.40%**	**Ni**	**32.20%**	**21.50%**	**Ni**	**32.20%**	**22.80%**
Si	894.0 ppm	0.13%	Si	0.85%	1.15%	Mg	0.47%	0.76%	Mg	0.96%	1.65%
Cl	1.13%	1.27%	P	1.01%	1.24%	Si	686.3 ppm	0.01%	Si	2.70%	4.00%
Ca	2.99%	2.98%	Ca	0.61%	0.58%	Ca	3.55%	3.5 ppm	P	0.84%	1.13%
Ti	182.5 ppm	153 ppm	Ti	57 ppm	45.4 ppm	Ti	25.2 ppm	20.6 ppm	Ca	1.62%	1.69%
Fe	391 ppm	280 ppm	Fe	351.6 ppm	240 ppm	Fe	255.8 ppm	179.4 ppm	Ti	96.7 ppm	84.1 ppm
Zn	237.3 ppm	145 ppm	Zn	0.20%	0.12%	Zn	0.18%	0.11%	Fe	0.10%	767.8 ppm
As	8 ppm	4.27 ppm	As	11.8 ppm	6 ppm	As	7.2 ppm	376.3 ppm	Zn	0.14%	872.2 ppm
Se	2.7 ppm	1.4 ppm	Se	1.7 ppm	0.8 ppm	Se	3.2 ppm	1.6 ppm	As	11.3 ppm	6.3 ppm
Br	65.9 ppm	33 ppm	Br	679.4 ppm	324 ppm	Br	57.8 ppm	28.3 ppm	Se	3.6 ppm	1.9 ppm
Sr	98.9 ppm	45.2 ppm	Sr	6.4 ppm	2.8 ppm	Sr	113.3 ppm	50.6 ppm	Br	6.6 ppm	3.4 ppm
Zr	14.9 ppm	6.5 ppm	Sn	138.8 ppm	44.6 ppm	Zr	19.3 ppm	8.3 ppm	Sr	19.3 ppm	9.2 ppm
Sn	162.2 ppm	54.7 ppm	Sb	164 ppm	51.4 ppm	Sn	104.3 ppm	34.4 ppm	Sn	203.8 ppm	71.5 ppm
Sb	65.7 ppm	21.6 ppm	Te	105.7 ppm	31.6 ppm	Sb	35.7 ppm	11.5 ppm	Sb	64.5 ppm	22.1 ppm
Te	140.8 ppm	44.2 ppm	I	661.9 ppm	199 ppm	Te	94.4 ppm	29 ppm	Te	179.0 ppm	58.4 ppm
I	132.5 ppm	41.8 ppm	Ba	30.4 ppm	8.5 ppm	Pb	16.5 ppm	3.1 ppm	Au	16.0 ppm	3.4 ppm
Re	0.5 ppm	0.1 ppm	Er	0.12%	274 ppm				Pb	2.3 ppm	0.5 ppm
Pb	9.4 ppm	1.8 ppm	Pb	13.3 ppm	2.5 ppm						

Fourier transform infrared and Raman spectroscopy

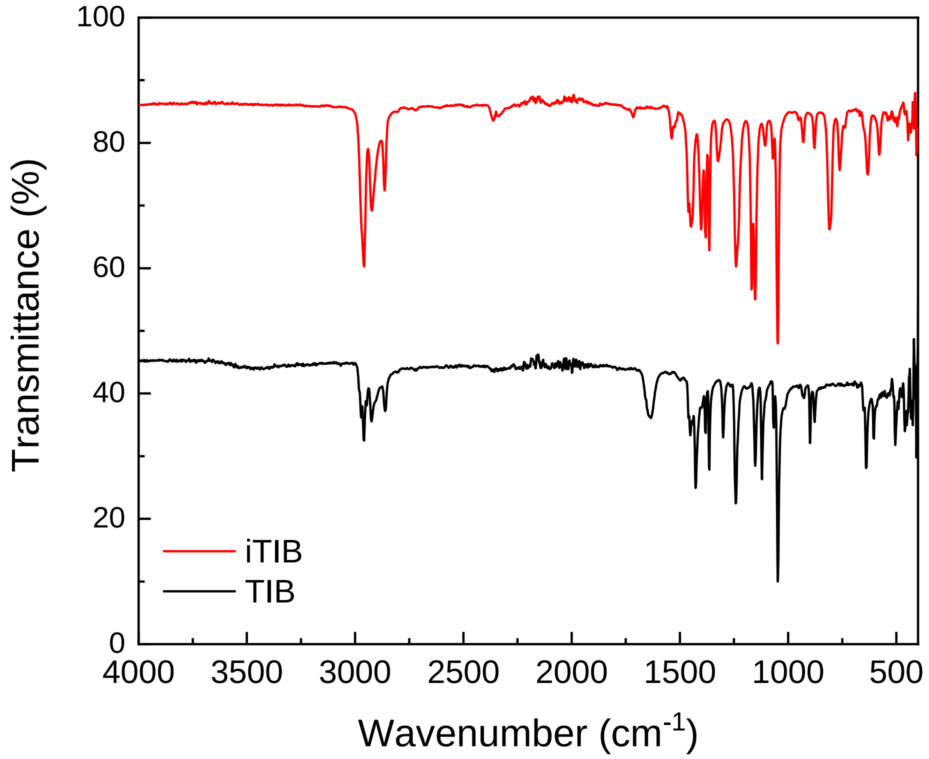

Figure S8. FTIR spectra of **TIB** and **iTIB**. The spectra have been stacked for clarity.

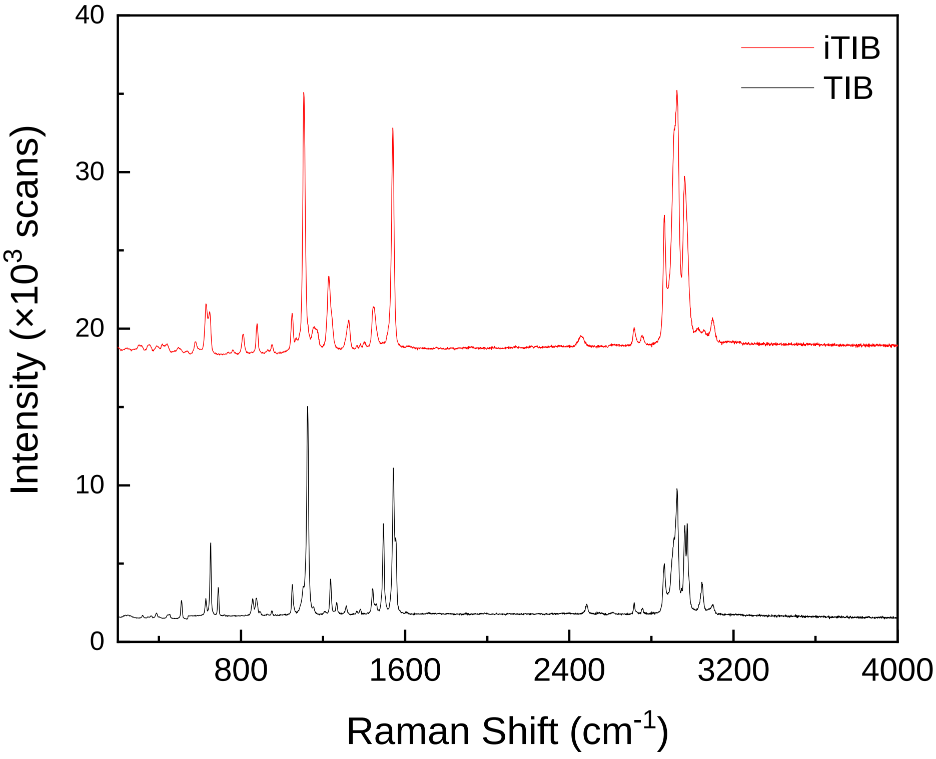

Figure S9. Resonance Raman (728 nm excitation) spectra of **TIB** and **iTIB**. The spectra have been stacked for clarity.

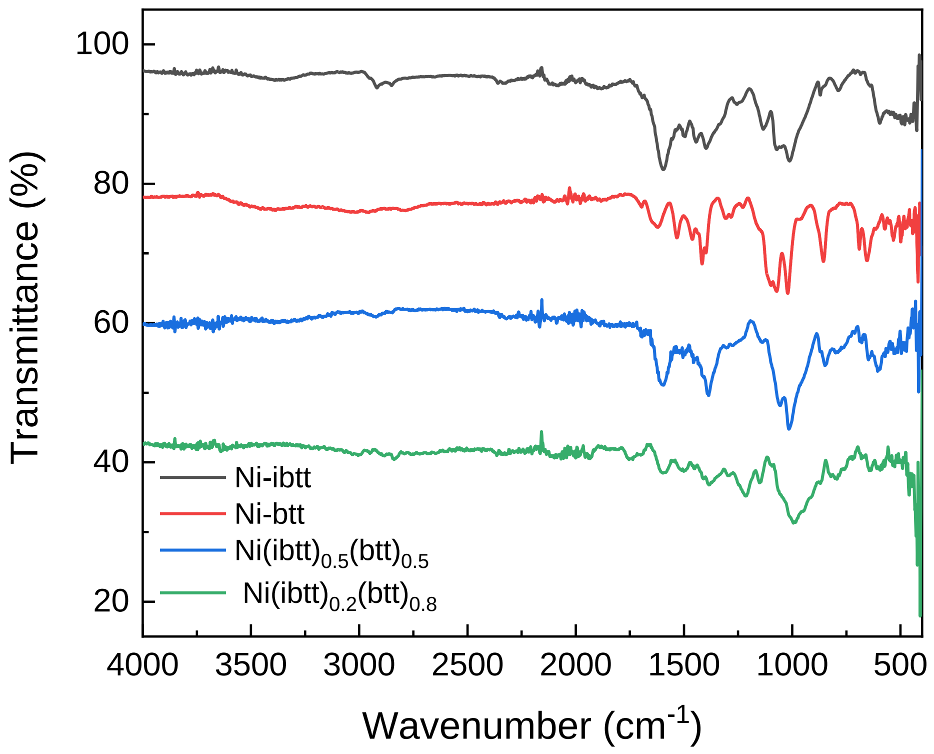

Figure S10. FTIR spectra of **Ni-btt**, **Ni-ibtt**, **Ni(ibtt)$_{0.5}$(btt)$_{0.5}$** and **Ni(ibtt)$_{0.2}$(btt)$_{0.8}$**. The spectra have been stacked for clarity.

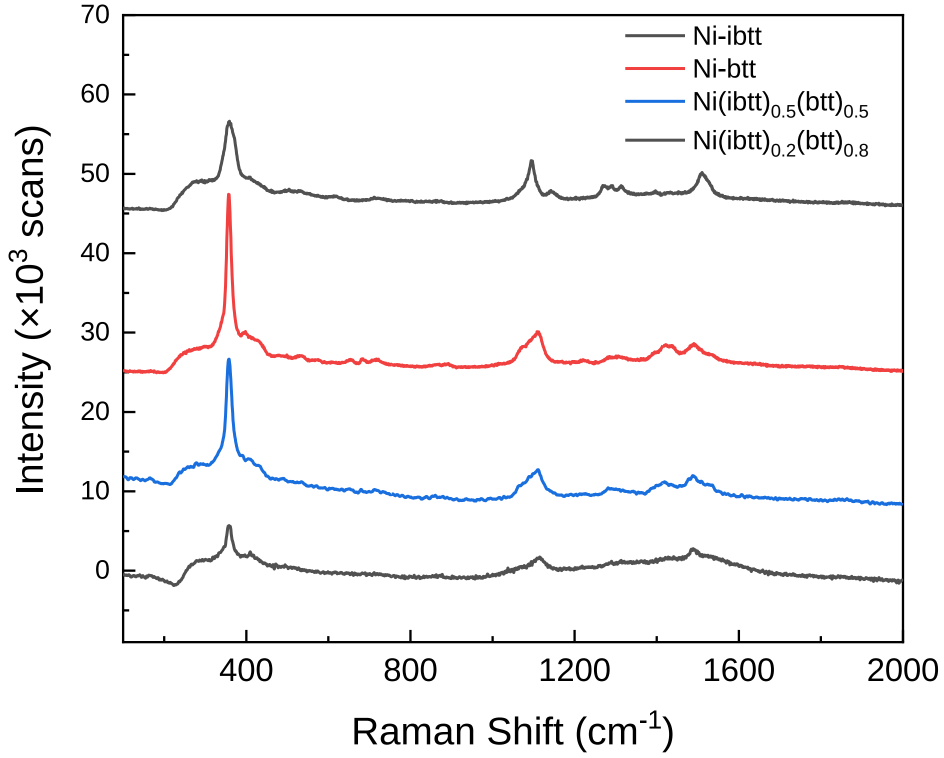

Figure S11. Resonance Raman (728 nm excitation) spectra of **Ni-btt**, **Ni-ibtt**, **Ni(ibtt)$_{0.5}$(btt)$_{0.5}$** and **Ni(ibtt)$_{0.2}$(btt)$_{0.8}$**. The spectra have been stacked for clarity.

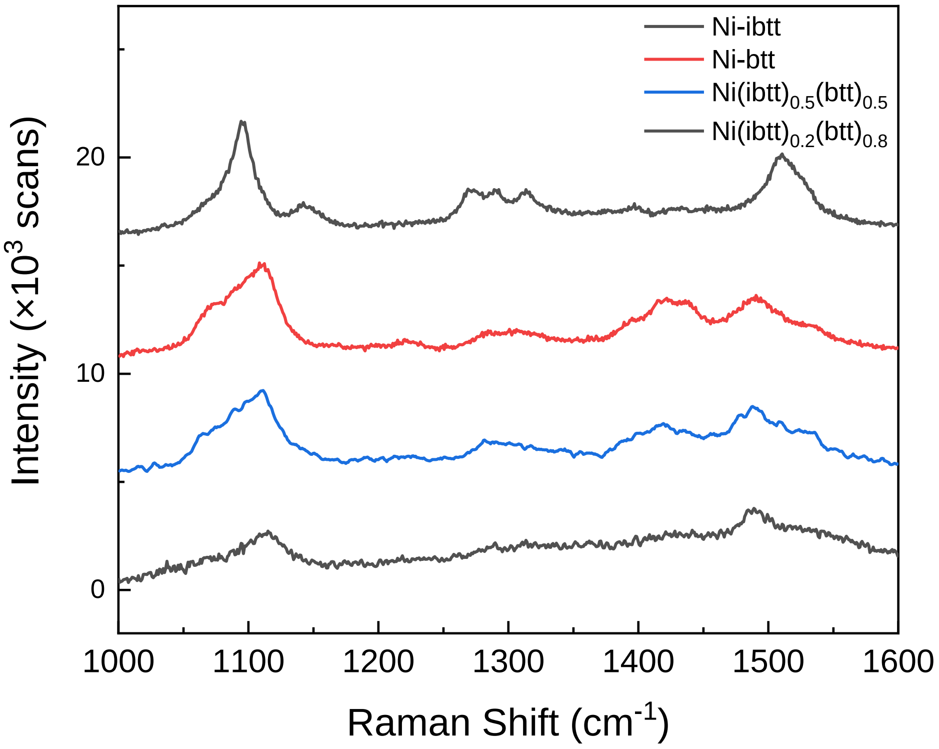

Figure S12. Resonance Raman (728 nm excitation) spectra of **Ni-btt**, **Ni-ibtt**, **Ni(ibtt)$_{0.5}$(btt)$_{0.5}$** and **Ni(ibtt)$_{0.2}$(btt)$_{0.8}$**. The spectra have been stacked for clarity.

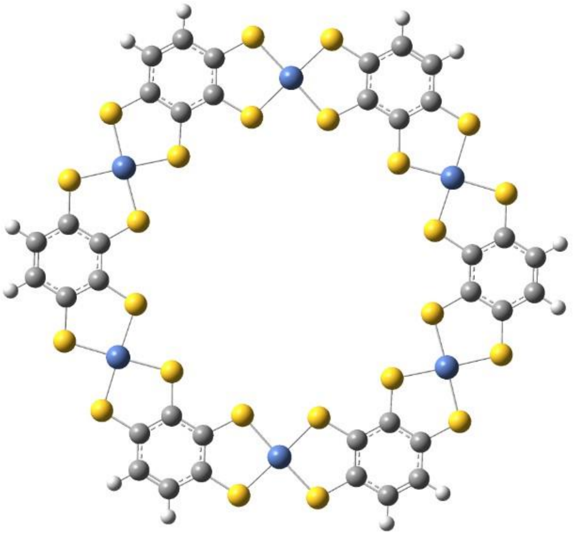

Figure S13. Minimum energy conformation obtained from DFT calculations (B3LYP/def2-SVP) for hexamers of **Ni-ibtt** with all metal-ligand complexes in the *cis*-configuration.

Powder X-ray diffraction

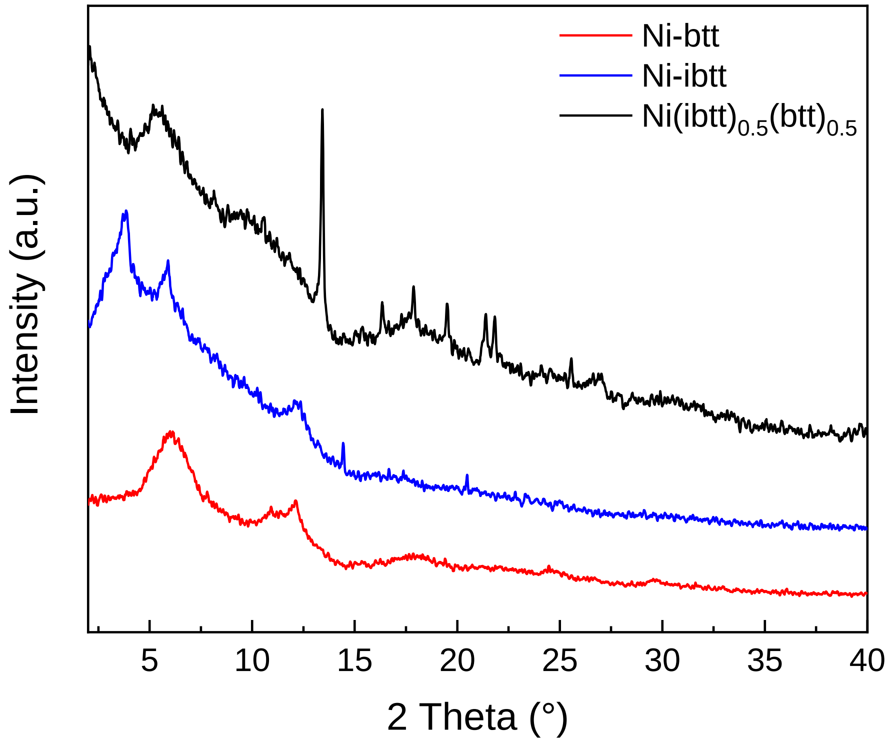

Figure S14. Powder x-ray diffractograms of **Ni-btt**, **Ni-ibtt** and **Ni(ibtt)$_{0.5}$(btt)$_{0.5}$**. The spectra have been stacked for clarity.

Thermogravimetric analysis

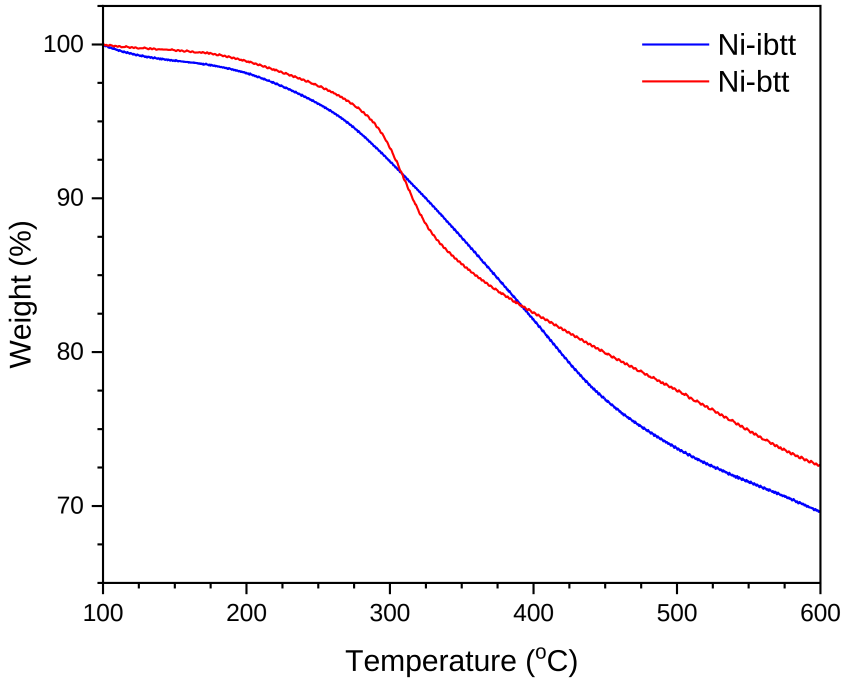

Figure S15. Thermogravimetric analysis data of **Ni-btt**, **Ni-ibtt**.

Electron paramagnetic resonance

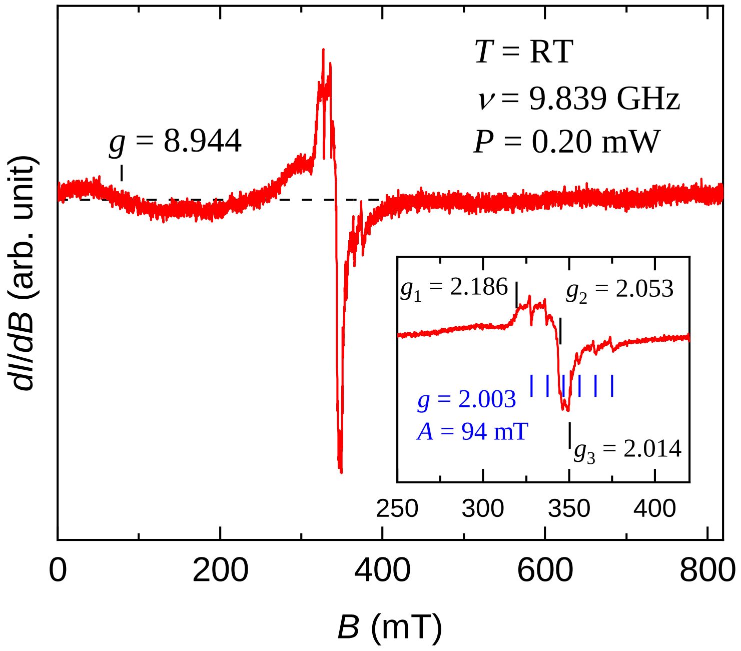

Figure S16. First derivative EPR spectra of **Ni-ibtt** recorded at 77 K.

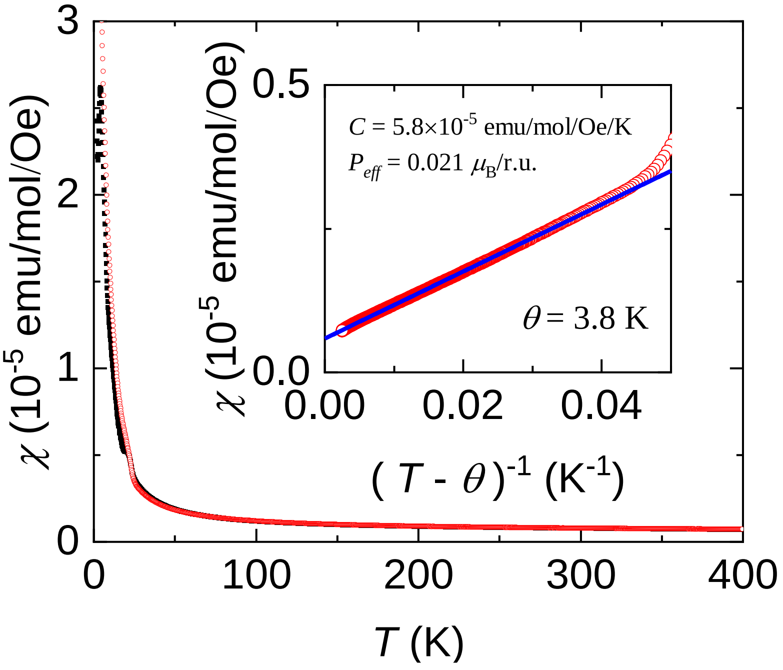

Figure S17. Temperature dependence of magnetic susceptibility (χm) of **Ni-ibtt** measured by SQUID.

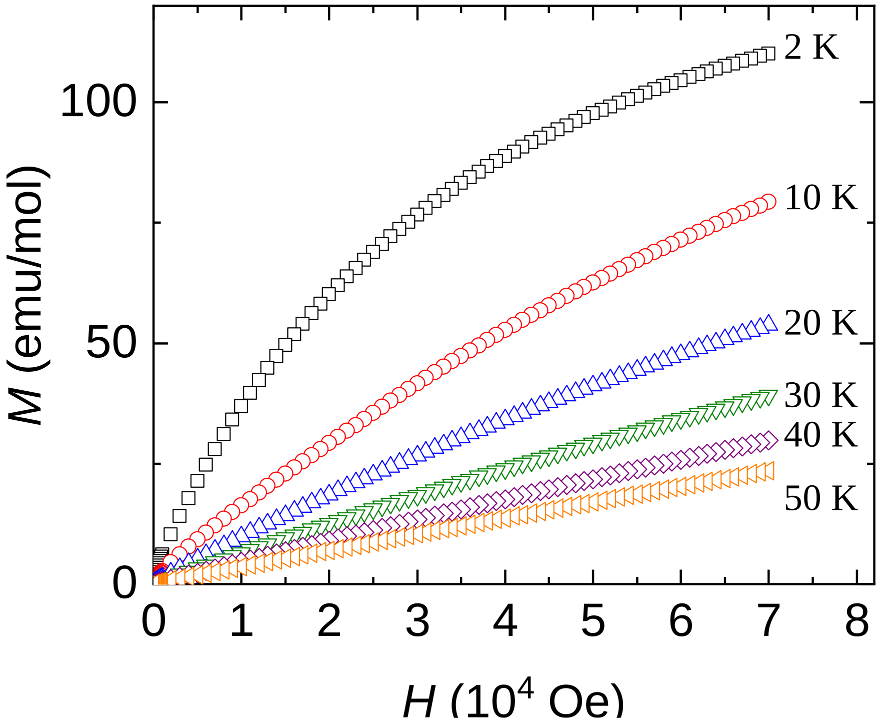

Figure S18. Magnetization of **Ni-ibtt** as a function of the applied magnetic field at selected temperatures.

Table S2. Summary of the EPR parameters for **Ni-ibtt**. μB is the Bohr magneton. r.u. is the repeat unit of the OMCP.

**SVSM**		**EPR**	
p_{eff} (μ_B/r. u.)	N_{spin} (/r. u.)*	N_{spin} (/r. u.)	
0.021	0.012	0.031 (central field)	g_1 = 2.186, g_2 = 2.053, g_3 = 2.014<br>Ni^{3+} LS (d^7, S = 1/2)<br>g ~ 2.003, A = 94 mT<br>Mn^{2+} HS (d^5, S = 5/2)
		0.021 (low field)	g ~ 8.9<br>Ni^{2+} HS (d^8, S = 1)

Density functional theory

Cluster Density Functional Theory (DFT) calculations we performed on oligomeric cluster models of the Ni-btt and 100%-trans Ni-ibtt coordination polymers. These models consisted of four nickel ions bridged by three benzene-1,2,4,5-tetrathiolate or benzene-1,2,3,4-tetrathiolate ligands, respectively, and capped with terminal benzene-1,2-dithiolate capping ligands, see Fig. S19, while we also performed calcations on their octomer equivalents with eight nickel atoms and nine ligands, see Fig. 4 in the main text. The octamer calculations were performed to get a general idea of the shape of the polymers and the tetramer oligomers were used for a full exploration of possible electronic states of the materials. The cluster calculations used a combination of the B3LYP hybrid functional, [1-4] incorporating 20% Hartree-Fock exchange, the Grimme's D3 dispersion correction[5] and the def2-SVP basis-set[6] and were performed using Turbomole 7.5.[7, 8] Multiple spin-states were explored for each oligomer while also calculations on cationic and anionic versions of the oligomers were performed to calculate the oligomers ionisation potential and electron affinity.

Periodic DFT calculations were performed on periodic models of the Ni-btt and 100%-trans Ni-ibtt coordination polymers using the Vienna Ab Initio Simulation Package (VASP).[9-12] These calculations use the projector augmented wave method, with scalar-relativistic pseudopotentials and 3p electrons included in the valence shell for Ni.[13] In line with our previous study on Ni coordination polymers,[14] the hybrid functional HSE06, incorporating 25% Hartree-Fock exchange and a screening parameter of 0.11 bohr^{-1} was used to describe the exchange-correlation interactions.[15, 16] The periodic models of the coordination polymers were constructed based the central part of the oligomeric models and consist of a single chain in one dimension (along c for Ni-btt, and a for Ni-ibtt), with a cell size of 30 Å in the other two dimensions to prevent interaction between periodic images. A plane wave energy cutoff of 600 eV and k-point meshes of 1 × 1 × 4 and 2 × 1 × 1 respectively were sufficient to converge the total energy of each polymer to within 1 meV per atom. Multiple initial spin states were trialled for each polymer, and the structures allowed to relax until the forces on each atom were below 0.01 eV Å^{-3}. The sumo package was used for plotting the electronic band structures,[17] with the high-symmetry path through the Brillouin zone taken from Bradley and Cracknell.[18] Curvature effective masses were obtained using a five-point parabolic fit to the band edges using the effmass package, and Kane dispersion parameters were calculated to assess non-parabolicity.[19]

Table S3. (S)HOMO and (S)LUMO energies and -IP and -EA values predicted for the oligomeric models of **Ni-ibtt** and **Ni-btt** (B3LYP/def2-SVP).

	**(S)HOMO**	**(S)LUMO**	**-IP**	**-EA**
**Ni-ibtt**	-5.82	-4.50	-6.47	-3.88
**Ni-btt**	-5.88	-4.90	-6.37	-4.47

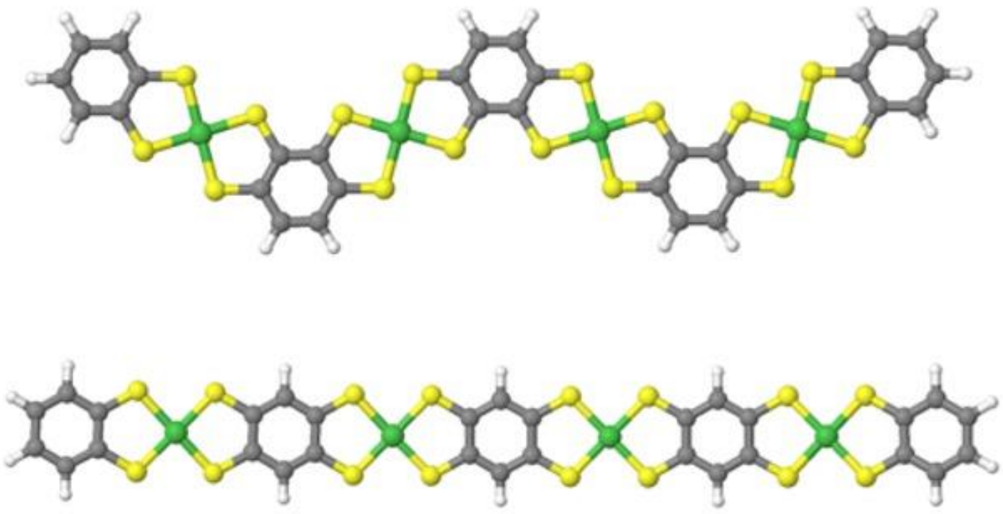

Fig. S19 Structures of the tetramer cluster models of Ni-ibtt, top, and Ni-btt, bottom, used in the cluster calculations.

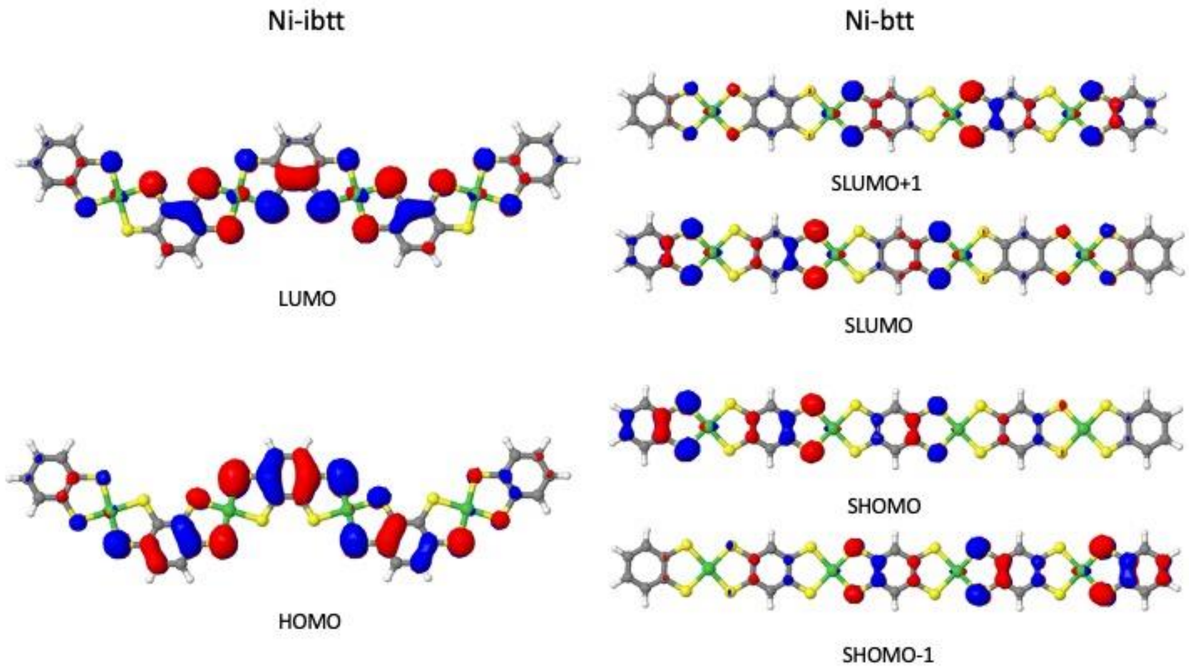

Figure S20. Frontier orbitals for the electronic ground state of **Ni-ibtt**, left, and **Ni-btt**, right. In the case of **Ni-btt** two occupied and two unoccupied orbitals are shown because the open-shell singlet ground state of **Ni-btt** is calculated with unrestricted DFT and thus spin-polarised molecular orbitals with one electron per orbital and not two as in the restricted calculations for closed-shell **Ni-ibtt**.

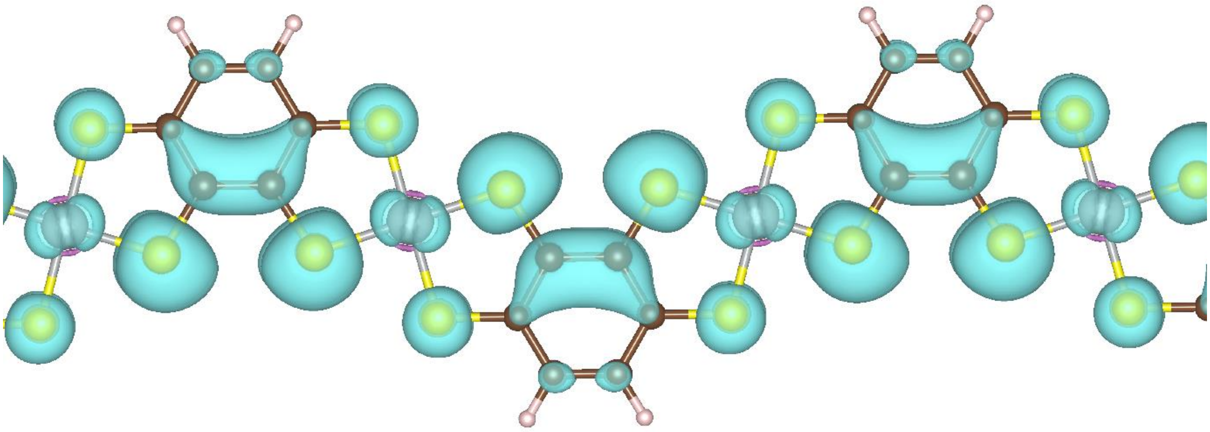

Figure S21. Partial charge density of the conduction band minimum (Γ) of 100%-trans **Ni-ibtt**, shown at an isosurface level of 6×10^{-4} e Å^3.

Thermoelectric characterisation

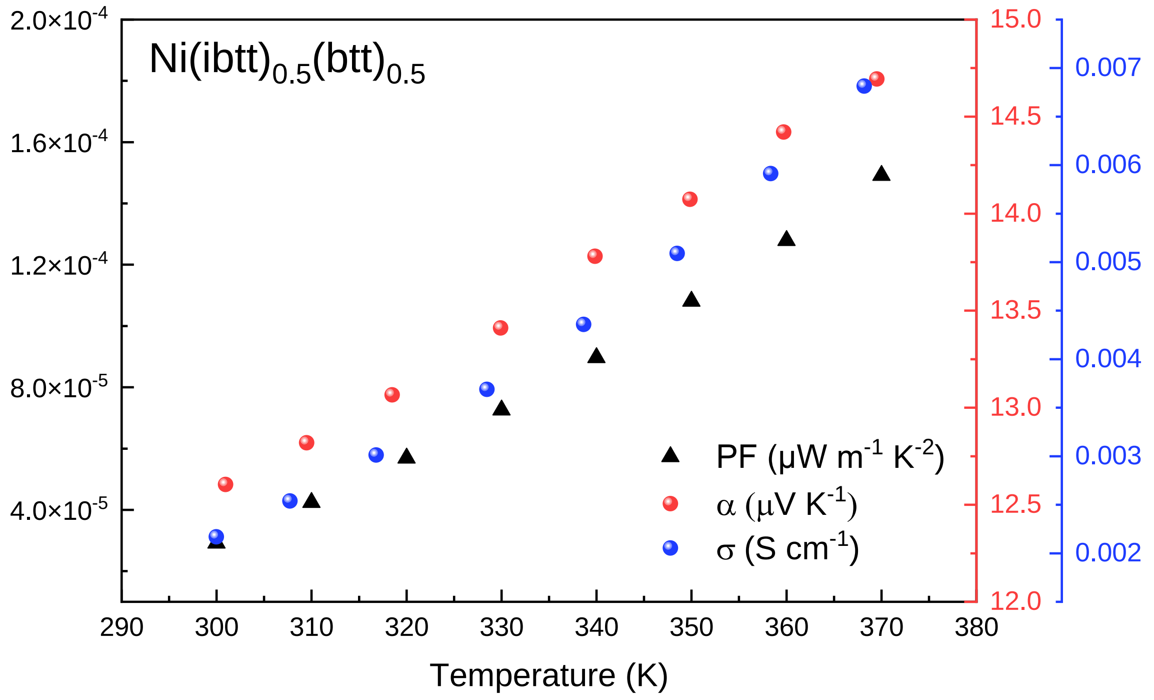

Figure S22. Thermoelectric properties of **Ni(ibtt)$_{0.5}$(btt)$_{0.5}$**, electrical conductivities (blue dots), Seebeck coefficients (red dots) and the power factor (black triangles).

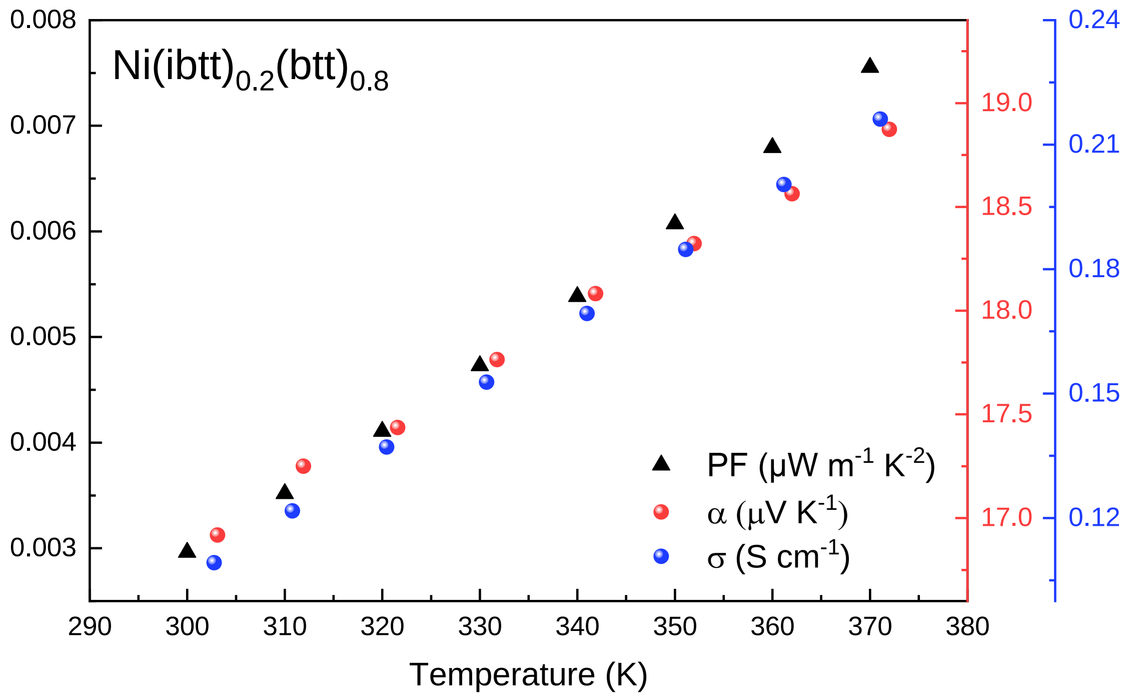

Figure S23. Thermoelectric properties of **Ni(ibtt)$_{0.2}$(btt)$_{0.8}$**, electrical conductivities (blue dots), Seebeck coefficients (red dots) and the power factor (black triangles).

References

1. Becke, A.D. Density-functional thermochemistry. III. The role of exact exchange. *The Journal of Chemical Physics* **98**, 5648-5652 (1993).
2. Lee, C., Yang, W. & Parr, R.G. Development of the Colle-Salvetti correlation-energy formula into a functional of the electron density. *Phys Rev B Condens Matter* **37**, 785-789 (1988).
3. Vosko, S.H., Wilk, L. & Nusair, M. Accurate spin-dependent electron liquid correlation energies for local spin density calculations: a critical analysis. *Canadian Journal of Physics* **58**, 1200-1211 (1980).
4. Stephens, P.J., Devlin, F.J., Chabalowski, C.F. & Frisch, M.J. Ab Initio Calculation of Vibrational Absorption and Circular Dichroism Spectra Using Density Functional Force Fields. *The Journal of Physical Chemistry* **98**, 11623-11627 (1994).
5. Grimme, S., Antony, J., Ehrlich, S. & Krieg, H. A consistent and accurate ab initio parametrization of density functional dispersion correction (DFT-D) for the 94 elements H-Pu. *J Chem Phys* **132**, 154104 (2010).
6. Weigend, F. & Ahlrichs, R. Balanced basis sets of split valence, triple zeta valence and quadruple zeta valence quality for H to Rn: Design and assessment of accuracy. *Physical Chemistry Chemical Physics* **7**, 3297-3305 (2005).
7. Furche, F. et al. Turbomole. *WIREs Computational Molecular Science* **4**, 91-100 (2014).
8. Balasubramani, S.G. et al. TURBOMOLE: Modular program suite for ab initio quantum-chemical and condensed-matter simulations. *The Journal of Chemical Physics* **152** (2020).
9. Kresse, G. & Hafner, J. Ab initio molecular dynamics for liquid metals. *Physical Review B* **47**, 558-561 (1993).
10. Kresse, G. & Hafner, J. Ab initio molecular-dynamics simulation of the liquid-metal--amorphous-semiconductor transition in germanium. *Physical Review B* **49**, 14251-14269 (1994).
11. Kresse, G. & Furthmüller, J. Efficiency of ab-initio total energy calculations for metals and semiconductors using a plane-wave basis set. *Computational Materials Science* **6**, 15-50 (1996).
12. Kresse, G. & Furthmüller, J. Efficient iterative schemes for ab initio total-energy calculations using a plane-wave basis set. *Physical Review B* **54**, 11169-11186 (1996).
13. Blöchl, P.E. Projector augmented-wave method. *Physical Review B* **50**, 17953-17979 (1994).
14. Liu, Z. et al. Controlling the Thermoelectric Properties of Organometallic Coordination Polymers via Ligand Design. *Advanced Functional Materials* **30**, 2003106 (2020).
15. Heyd, J., Scuseria, G.E. & Ernzerhof, M. Hybrid functionals based on a screened Coulomb potential. *The Journal of Chemical Physics* **118**, 8207-8215 (2003).
16. Janesko, B.G., Henderson, T.M. & Scuseria, G.E. Screened hybrid density functionals for solid-state chemistry and physics. *Phys Chem Chem Phys* **11**, 443-454 (2009).
17. M Ganose, A., J Jackson, A. & O Scanlon, D. sumo: Command-line tools for plotting and analysis of periodic ab initio calculations. *J. Open Source Softw.* **3**, 717 (2018).
18. Bradley, C. & Cracknell, A.P. The mathematical theory of symmetry in solids. (Oxford University Press, London, England; 2009).
19. D. Whalley, L. effmass: An effective mass package. *J. Open Source Softw.* **3**, 797 (2018).
